# Identification of molecular clusters and a risk prognosis model for diffuse large B-cell lymphoma based on lactate metabolism-related genes

**DOI:** 10.1007/s00277-025-06321-1

**Published:** 2025-04-05

**Authors:** Jie Zhang, Ting Gao, Shan Chen, Shuang Wu, Yong Mao, Dongyan Cai, Tingxun Lu

**Affiliations:** 1 Wuxi School of Medicine, Jiangnan University, Wuxi, Jiangsu Province 214122 China; 2 Department of Oncology, Affiliated Hospital of Jiangnan University, No.1000, Hefeng Road, Wuxi, Jiangsu Province 214122 P.R. China; 3 Department of Gastroenterology, The Fourth People’s Hospital of Sichuan Province, Chengdu, Sichuan Province 610020 China; 4 Department of Hematology, Affiliated Hospital of Jiangnan University, Wuxi, Jiangsu Province 214122 China

**Keywords:** DLBCL, Lactate metabolism, Prognosis model, Immune microenvironment

## Abstract

Diffuse large B-cell lymphoma (DLBCL) is a leading cause of morbidity and mortality among lymphomas in adults, with tumor cells undergoing metabolic reprogramming linked to the immune microenvironment. This study explored the relationship between lactate metabolism-related genes (LMRGs), DLBCL prognosis, and immune microenvironment interactions. Publicly available datasets (GSE10846 and GSE87371) were analyzed, with LMRGs identified using Cox regression and LASSO regression. A risk prognosis model comprising five LMRGs was developed, showing that high-risk patients had worse outcomes due to adverse clinical features, aggressive immune microenvironments, and poor treatment responses. A nomogram combining the model with clinical data predicted 1-, 3-, and 5-year survival. Single-cell RNA sequencing indicated that high LMRG risk scores in B cells may promote immunosuppression via the MIF-CD74/CXCR4 pathway. Functional validation revealed that SDHA knockdown reduced DLBCL cell proliferation in U2932 and KIS-1 cell lines. This LMRG-based model serves as a valuable tool for predicting survival, immune landscape, and clinical risk stratification in DLBCL patients, while also highlighting the crucial role of lactate metabolism in DLBCL pathogenesis. Furthermore, these findings underscore the potential of LMRGs risk scores to guide personalized therapies and improve treatment outcomes.

**Supplementary Information:**

The online version contains supplementary material available at 10.1007/s00277-025-06321-1.

## Introduction

Diffuse large B-cell lymphoma (DLBCL), the most common histological subtype of non-Hodgkin lymphoma, exhibits considerable heterogeneity in therapeutic response and prognosis [1]. Notwithstanding that rituximab plus conventional therapy salvage about 60% of patients, a significant challenge remains with relapsed and refractory disease occurring in around 30–40% of cases [2]. Current stratification heavily relies on the International Prognostic Index (IPI) and other optimized versions of IPI. However, due to the underlying heterogeneity of the disease severity, these staging systems have limited predictive power [3]. Further research is needed to optimize stratification models with novel biomarkers to guide poor outcomes.

Not surprisingly, acidity results from tumor metabolism, particularly through the process of aerobic glycolysis. This phenomenon was first observed by Otto Warburg, who named it the Warburg effect, and it currently acts as a central metabolism hallmark of cancer cells [4]. Otto Warburg presented this form of metabolic reprogramming in the presence of the precondition of mitochondria dysfunction. Carbons initially enter lactate metabolism from pyruvate and subsequently shunt into mitochondrial metabolism. This metabolic pathway proves to be nonredundant in generating ATP and biosynthetic precursors within cancer cells [5, 6]. Lactate is a metabolic fuel for cancer cells [7]. Cancer cells can absorb the extracellular lactate through the monocarboxylate transporter 1 (MCT1) and convert it to pyruvate, then enter into the tricarboxylic acid (TCA) cycle to generate ATP [8]. Moreover, Marta Braga et al. also demonstrated that lactate is used as a metabolic fuel rather than the waste product of glycolysis in DLBCL cells [9].

Although, up to now, immunotherapy has been successful only in a subset of patients, applying the power of the immune system has immense potential in tumor control. With the advent of several technological progressions, a growing focus has been on the role of lactic acid and lactate in the tumor microenvironment (TME) [7]. TME is a unique metabolic niche composed of extracellular matrix, immune cells, stromal cells, and cancer cells. Notably, it is essential to maintain the homeostasis of both physicochemical properties and constituent elements, especially the physiological acid concentration surrounding the tumor and immune cells. However, the extracellular accumulation of lactic acid and lactate was produced by tumor cells and bystander cells, such as cytotoxic CD8^+^T cells [10], regulatory T cells (Tregs) [11], and macrophages [12], leading to a scenario where immune cell-intrinsic functions and their interactions with tumor cells became ineffective in the tumor milieu [7]. This accumulation not only impaired antitumor immunity but also served as a metabolic resource that supported tumor growth by fueling tumor cells. Therefore, targeting lactate and lactate-related metabolic pathways represented a promising therapeutic strategy to mitigate tumor progression by restoring immune efficacy and reducing nutrient availability for tumor cells.

Herein, we screened several lactate metabolism-related genes (LMRGs) in a lactate metabolism-related gene set and identified them with prognostic value. Using gene expression and clinical information from the GSE10846 dataset, we constructed an LMRGs-based risk prognosis model, which was subsequently validated in the GSE87371 dataset. Our study delved into the potential correlations between the risk model and clinicopathological features, immune profile, and treatment responses. Single-cell analysis revealed cell interactions based on the LMRGs risk score. Additionally, we elucidated the biological characteristics of SDHA, a representative LMRG, in DLBCL. The findings from these analyses elucidated the critical role of LMRGs and immune cells within the TME, emphasizing the promising research value of LMRGs in the pathogenesis and potential therapy of DLBCL.

## Materials and methods

### Data sources

Gene expression profiles and corresponding clinical data (GSE10846 and GSE87371) of DLBCL patients were acquired from the gene expression omnibus (GEO) database (https://www.ncbi.nlm.nih.gov/geo/*).* The Affymetrix Human Genome U133 Plus 2.0 Array used tumor tissues from these two databases. In GSE10846, we downloaded the raw data and adopted the GCRMA method to normalize gene expression as advocated [13]. Data processing of GSE87371 was analyzed with GeneSpring software (Agilent Technologies) using default settings and the RMA normalization and summarization algorithm. Unavailable or unknown clinical survival information was excluded. Then, 412 DLBCL patients in GSE10846 and 221 DLBCL patients in GSE87371 were designed as training and validation groups, respectively. Relevant grouping information and clinicopathological features are shown in Table S1. In processing chip annotation, we extracted each gene corresponding to each probe and selected the average of multiple genes for consolidation. Publicly available scRNA-seq data from Roider and colleagues [14] was accessible via the heiDATA database at 10.11588/data/VRJUNV. This dataset includes single-cell RNA sequencing data from 3 DLBCL samples and 3 reactive lymph node (rLN) samples.

Additionally, LMRGs were obtained from previously published studies, where a set of 294 LMRGs were retrieved from the Molecular Signatures Database (MSigDB, https://www.gsea-msigdb.org/gsea/msigdb*).* Fifteen LMRGs have been involved in constructing lactate metabolism-related models in other tumors. After calculating the union with the genes in the database, 309 LMRGs were included in this study, as shown in Table S4.

### Identification of the prognostic LMRGs and clustering analysis of LMRGs

To screen the LMRGs with predictive value further, we employed univariate COX regression analysis to narrow down the number of LMRGs. After 98 LMRGs stood out, the ConsensusClusterPlus R package classified patients in the training dataset into two groups based on these genes. Cox regression (univariate and multivariate) and least absolute shrinkage and selection operator (LASSO) regression were performed to filter remarkable prognostic genes from the previously recognized LMRGs. We set *p* < 0.05 as the threshold for univariate Cox regression analysis to preliminarily select prognostic LMRGs. Subsequently, for the LASSO-Cox analysis, the threshold for univariate Cox regression was set more leniently at *p* = 0.1, while the threshold for multivariate regression Cox remained at *p* < 0.05. Moreover, LASSO estimation, adjusting the penalty parameter λ using the glmnet R package [15], allowed non-significant gene coefficients to be shrunk to 0, retaining only the genes essential for model prediction.

### Construction and assessment of the LMRGs-related risk prognosis model

In addition, the coefficients of prognostic LMRGs obtained in multivariate COX regression were multiplied by the corresponding gene expression levels to construct a default prognostic scoring formula. After that, each DLBCL patient got their risk score based on the above measure. The median LMRGs risk score in the training group was a rational cut-off value to divide DLBCL patients into high- and low-risk groups, which also applied to the validation group. Using the principal component analysis (PCA) could visualize the classification of DLBCL patients dependent on LMRGs risk scores. Survival analysis was presented by the Kaplan-Meier curve to show the prognosis difference between high- and low-risk groups, and the *p* value labeled in the figure was calculated using the log-rank test.

Meanwhile, we separately evaluated the predictive effects of the LMRGs risk scores in DLBCL populations with different cell origin classifications. Furthermore, multiple bioinformatics methods assessed the predictive utility of the risk prognosis model. A time-dependent receiver operating characteristic (ROC) curve analysis was used to evaluate the model prediction performance and the area under the curve (AUC) of survival by the timeROC R package.

### Construction and evaluation of the nomogram

The nomogram was developed to individualize the predicted survival possibility for 1-, 3-, and 5-year by rms R package [16]. This study incorporated eight variables into the nomogram: age, gender, Eastern Cooperative Oncology Group Performance Status (ECOG PS), Ann Arbor stage, IPI, number of extranodal sites, and risk score. Calibration and discrimination are the most frequently employed methods for evaluating the model’s performance. Here, the calibration curves were assessed graphically by plotting the nomogram-predicted probabilities against the actual possibilities, and the 45º line represented the optimal predictive values.

### Correlation analysis of risk prognosis model with immune landscape

The Cell-type Identification By Estimating Relative Subsets Of RNA Transcripts (CIBERSORT) algorithm is used to assess the immune infiltration levels of 22 subsets of human immune cells and visualized with the IOBRR package [17, 18]. Single-sample Gene Set Enrichment Analysis (ssGSEA) is executed to quantify 28 types of immune cells in tumor samples via the GSVA R package [19]. Estimation of Stromal and Immune cells in Malignant Tumors using Expression data (ESTIMATE) method is applied for the prediction of tumor purity and the content of stromal and immune cells from expression data, as well as ESTIMATE score for the immune environment [20].

### Correlation analysis of risk prognosis model with drug sensitivity and therapeutic response

The OncoPredict R package was conducted to predict clinical drug response in DLBCL patients. Using the core function “calcPhenotype” from the R package, the GDSC version V2 dataset was utilized as the training set, which includes the gene expression matrix and half-maximal inhibitory concentration (IC50) data for various drugs across multiple cancer cell lines. The GDSC2_Expr and GDSC2_Res datasets were downloaded from https://osf.io/c6tfx/, and the expression data were normalized to TPM (Transcripts Per Million) to ensure consistency. The GSE10846 dataset served as the test set, with its expression matrix processed in the same standardized format. The calcPhenotype function was then applied to predict drug sensitivity, generating IC50 values for each sample in the test set. Next, the tumor immune dysfunction and exclusion (TIDE) algorithm was applied to assess the potential response to immunotherapy in DLBCL patients.

### Differentially expressed genes analysis and enrichment analysis

We performed the limma R package to obtain differentially expressed genes (DEGs) between the two binary groups, “cluster” and “risk group.” Comparison matrices were created using the “makeContrasts” function for “cluster1 VS cluster2” and “high-risk group VS low-risk group.” The thresholds were set as follows: *p* value < 0.05 and absolute log2 fold change (|log2(FC)|) > 1. Differential genes were further analyzed for gene ontology (GO) analysis [21], including cellular component (CC), molecular function (MF), and biological process (BP), using the clusterProfiler R package [22], and the results were visualized.

### Cell culture

GM12878 (normal B cell line), U2932, KIS-1, RIVA, FARAGE, and CTB1 cell lines were cultured in 1640 medium (Bio-Channel) supplemented with 10% fetal bovine serum. Cells were maintained in appropriate media as recommended, under conditions of 37 °C and 5% CO2.

### QRT-PCR

Total RNA was extracted using TRIzol reagent (Invitrogen; Thermo Fisher Scientific, Inc.), and complementary DNA was synthesized with a reverse transcription kit (VazymeR323-01). qPCR was performed using a SYBR Green PCR kit (VazymeQ712-02) following the manufacturer’s instructions. GAPDH was used as an internal control. All qRT-PCR primers were designed with Primer Premier 5.0 and synthesized by Sangon. The primers of MYC, SDHA, and GAPDH are as follows: MYC forward (5’-CTCACAGCCCACTGGTCCTCAA-3’), reverse (5’-GACCCTCTTGGCAGCAGGATAG-3’); SDHAforward (5’-GACTACAAGGTGCGGATTGATGAG-3’), reverse (5’-GTGCTTCCTCCAGTGCTCCTC-3’); GAPDH forward (5’-AGAAGGCTGGGGCTCATTTG-3’), reverse (5’-AGGGGCCATCCACAGTCTTC-3’).

### Western blot

DLBCL cells were lysed in RIPA buffer with ice-cold PMSF to extract total proteins. Protein concentration was measured using the BCA assay. Proteins were separated on 10% SDS-PAGE gels and transferred to PVDF membranes. Membranes were blocked with 5% skimmed milk for 2 h at room temperature or overnight at 4˚C. Primary antibodies were diluted as instructed and incubated with the membranes at 4˚C overnight. After washing with TBST, membranes were incubated with HRP-conjugated secondary antibodies at 37˚C for 2 h. Protein bands were visualized using chemiluminescence (ChemiScope 5300 Pro). All antibodies utilized for Western blot analysis are detailed in Table S5.

### Immunohistochemistry (IHC)

This study included 5 normal tissue specimens and 18 DLBCL cases, all of which were formalin-fixed and paraffin-embedded (FFPE).The FFPE tissue sections were deparaffinized and subjected to antigen retrieval by heating in citrate buffer at 100 °C for 15 min. Endogenous peroxidase activity was blocked by incubating the sections in 3% hydrogen peroxide for 10 min. To reduce nonspecific binding, the sections were incubated with 5% bovine serum albumin (BSA) at room temperature for 30 min. Primary antibodies (Table S5) were applied to the sections and incubated overnight at 4 °C, followed by incubation with appropriate secondary antibodies at room temperature for 1 h. The sections were then developed using 3,3’-diaminobenzidine (DAB) and counterstained with hematoxylin for 3 min. After rinsing with running water, the sections were dehydrated, mounted, and examined under a microscope.

### Cell transfection

Following the reagent manufacturer’s protocol, two cell lines (U2932 and KIS-1) with high target gene expression were chosen for transient transfection. Transfection efficiency was evaluated by qRT-PCR. The sequences of siRNA are provided in Table S6.

### CCK-8

DLBCL cells (2 × 10⁴) were plated onto 96-well plates with 100 µL of cell suspension per well and cultured at 37˚C with 5% CO2. Cells were incubated with 10 µL of Cell Counting Kit-8 (CCK-8) solution (Beyotime) for 1–4 h in the dark. Absorbance (OD value) was measured, and cell viability was calculated based on the OD value.

### Analysis of the single-cell dataset

Single-cell sequencing is a technology used to study the genomics of individual cells. In this study, we utilized the Seurat package (v5) in R to convert single-cell sequencing data into Seurat objects, followed by preprocessing and normalization based on the standard workflow (https://github.com/satijalab/seurat/). The filtering criteria for cells were as follows: cells with more than 6000 genes or fewer than 300 genes were excluded, as were cells with > 10% mitochondrial genes and > 5% erythrocyte genes. Only genes expressed in at least three cells were included. Dimensionality reduction and clustering were performed after batch correction using the “HarmonyIntegration” method. Cell clusters were annotated based on marker genes specific to immune cells. The LMRGs risk scores for all cells were calculated based on the previously defined formula, and B cells and T cells were divided into high LMRGs signature group and low LMRGs signature group based on the median risk score. Finally, cell-cell communication analysis was conducted for each type of immune cell.

### Statistical analysis

All statistical analyses and plots were produced using R (v.4.2.2). Univariate and multivariate Cox regression analyses were performed to find the independent prognostic factors, including predictive genes and clinical variates. The point end for patients was overall survival (OS). The survival analysis was performed using the Kaplan-Meier estimate with 95% confidence intervals and log-rank test in separate curves. ROC analysis, a commonly utilized method, was used to assess the specificity and sensitivity of predictive efficiency based on the risk prognosis model. The AUC was used for quantitatively evaluating the accuracy of a model, and the AUC value greater than 0.7 was considered acceptable.

Wilcoxon rank-sum and Kruskal-Wallis tests were used to assess differences in risk scores across clinical-pathologic parameters (for two-group and multi-group comparisons, respectively). Associations between clinical characteristics and clustering/risk stratification were evaluated using Pearson’s Chi-squared test and Fisher’s exact test. The total hypothetical tests were two-sided, and *p* values less than 0.05 were prerequisites for statistical significance of differences in all present tests (*p < 0.05, ** *p* < 0.01, *** *p* < 0.001, and **** *p* < 0.0001).

## Results

### Identifying lactate metabolism-associated molecular subtypes and comprehensive analysis of differences in prognosis between subtypes

**Fig. 1:**
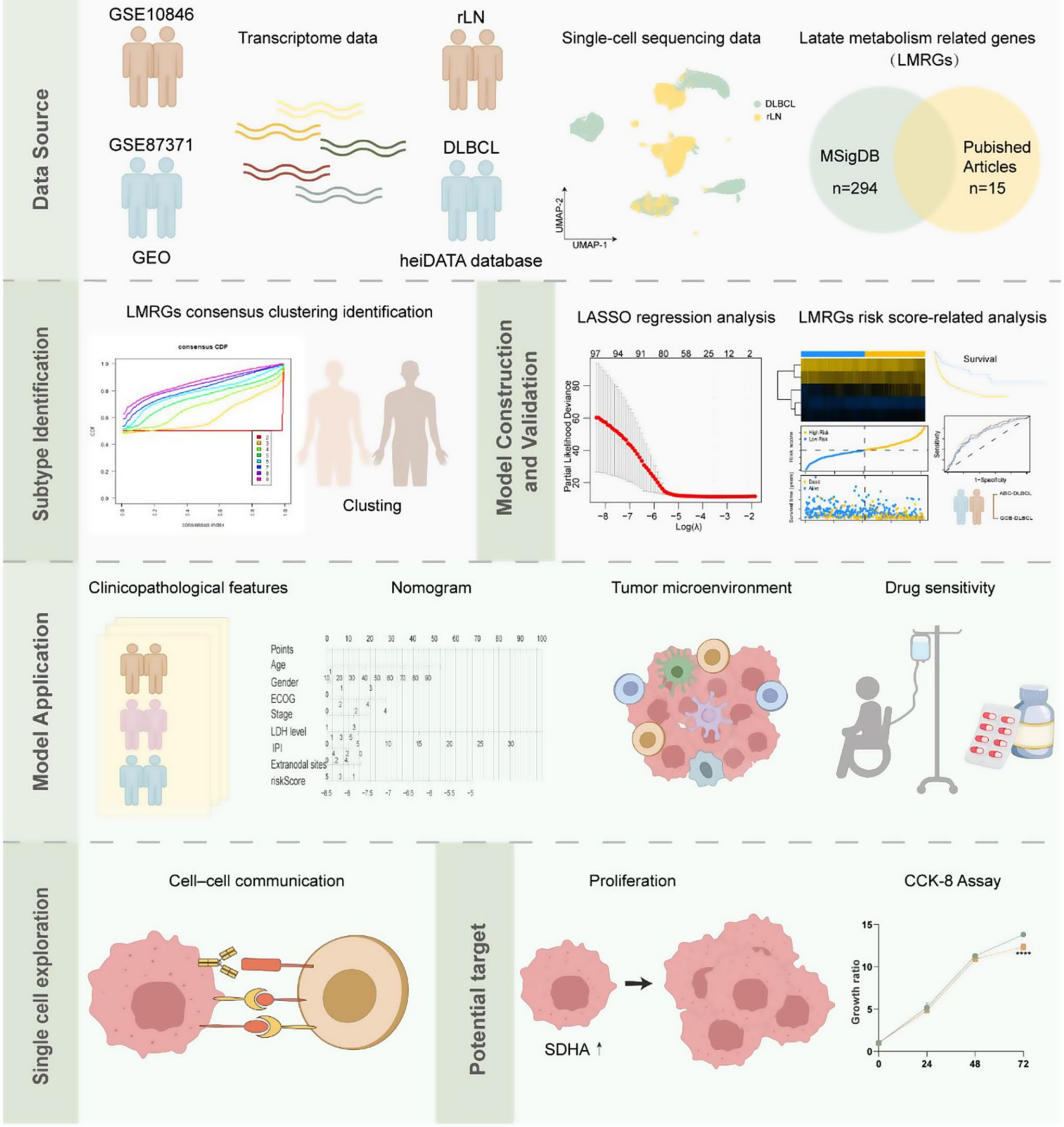
The workflow of this study. Abbreviations: GEO: gene expression omnibus; rLN: reactive lymph node; DLBCL: diffuse large B-cell lymphoma; MsigDB: molecular signatures database; LASSO: least absolute shrinkage and selection operator

**Fig. 2:**
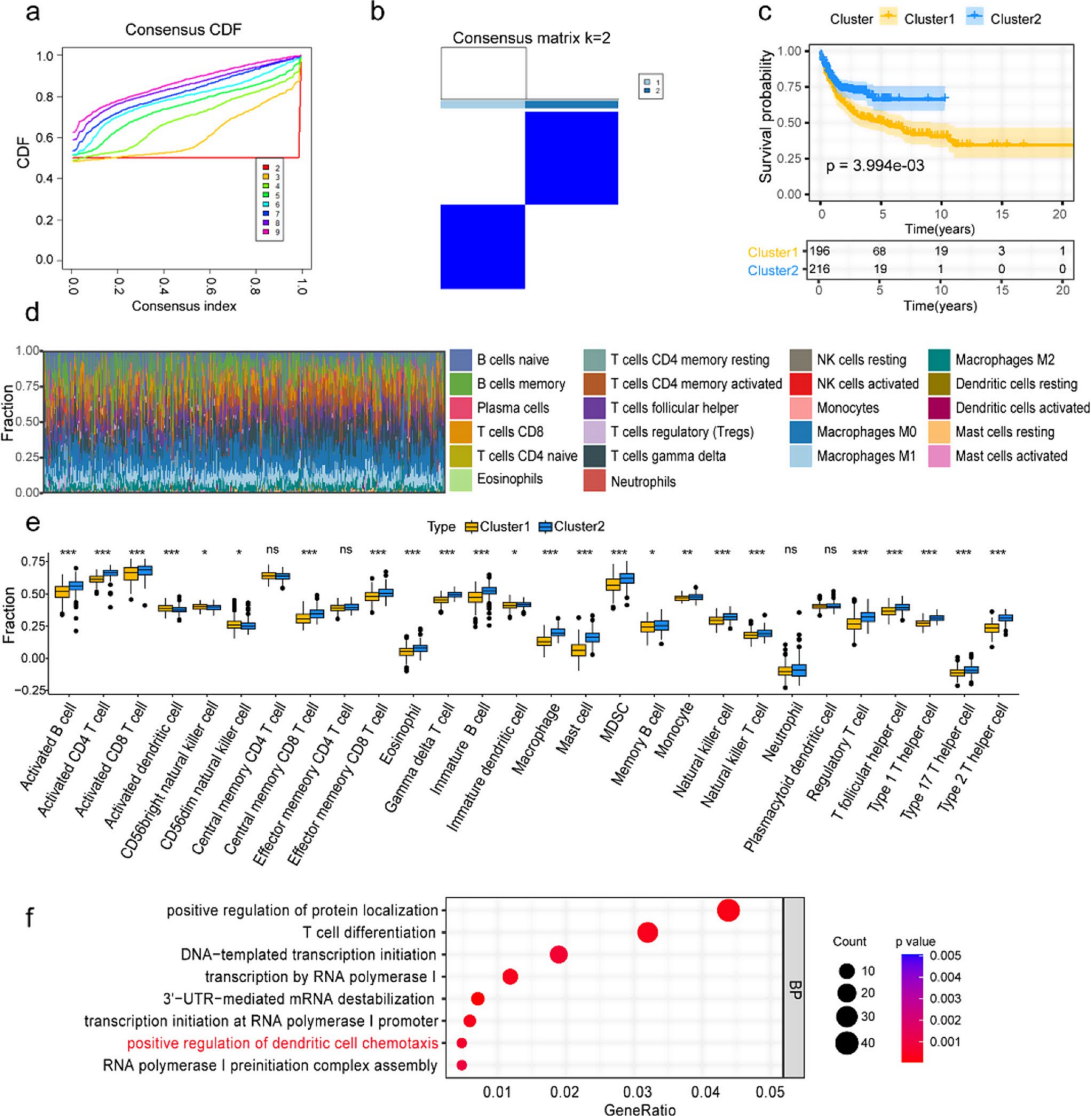
Consensus clustering identified two subtypes of LMRGs in GSE10846. **a** The CDF curves for clusters at k value 2 to 9. **b** Consensus clustering heatmap with k = 2. **c** Survival curve of patients in cluster 1 and cluster 2. **d** Immune cell fractions of DLBCL patients in GSE10846 evaluated via the CIBERSORT algorithm. **e** The differences in the immune infiltration landscape between cluster 1 and cluster 2 by the ssGSEA. **f** GO-BP analysis of upregulated differentially expressed genes in cluster 1. The “ns” indicates no significant difference

Figure 1 illustrates the graphical abstract of this study. First, we performed a univariate Cox regression analysis on the GSE10846 dataset, identifying 57 risk LMRGs and 41 protective LMRGs (Fig. S1a, b). We employed consensus clustering analysis to better understand the traits and distribution situation of these 98 LMRGs. After resampling, the optimal k was determined to be 2, at which point the slope of the Cumulative Distribution Function was minimal (Fig. 2a, b), implying that the two clusters have been well distinguished, as detailed in Table S2 which summarizes the distinct clinical features between clusters 1 and 2. Notably, the Kaplan-Meier curve indicated that cluster 1 had a shorter survival time and worse outcome than cluster 2 (*p* = 0.004; Fig. 2c). Then, to investigate immune-related mechanisms underlying prognostic differences, we performed comprehensive immune infiltration analyses. Using the CIBERSORT algorithm, we characterized immune cell proportions in GSE10846 patients (Fig. 2d). ssGSEA revealed reduced overall immune infiltration in cluster 1 compared to cluster 2, though activated dendritic cells (*p* < 0.001), CD56 bright NK cells (*p* < 0.05), and CD56 dim NK cells (*p* < 0.05) showed relative enrichment among 28 immune subtypes (Fig. 2e). Notably, genes upregulated in cluster 1 were enriched in dendritic cell chemotaxis regulation, suggesting that while these tumors exhibit overall immunosuppression, they may selectively enhance chemokine signaling to recruit specific DC subsets (Fig. 2f). Cluster 1 exhibited elevated expression of immunosuppressive checkpoints including CD244 (*p* < 0.001), CD274 (*p* < 0.01, PD-L1), CSF1R (*p* < 0.01), CTLA4 (*p* < 0.001), HAVCR2 (*p* < 0.001, TIM-3), LGALS9 (*p* < 0.001), and TGFB1 (*p* < 0.001, Fig. S1c). ESTIMATE analysis demonstrated significantly higher tumor purity (*p* < 0.001, Fig. S1d) alongside lower stromal (*p* < 0.001, Fig. S1d), immune (*p* < 0.001, Fig. S1d), and ESTIMATE scores in cluster 1 (*p* < 0.001, Fig. S1d), suggesting an immune-evasive phenotype dominated by tumor cells with compromised immune infiltration.

Furthermore, we focused on the differential LMRGs upregulated in cluster 1. The GO-BP analysis revealed that these genes mainly concentrated in the cellular response to oxygen levels or hypoxia, the glucose metabolic process, the hexose metabolic process, the monosaccharide metabolic process, and the phosphatidylserine metabolic process (Fig. S1e). Therefore, these findings suggested that the distinctions in immune landscape and response to metabolism and oxygen tension may affect the prognosis between these two subtypes.

### Construction of the LMRGs-based risk prognosis model with validity assessment

**Fig. 3:**
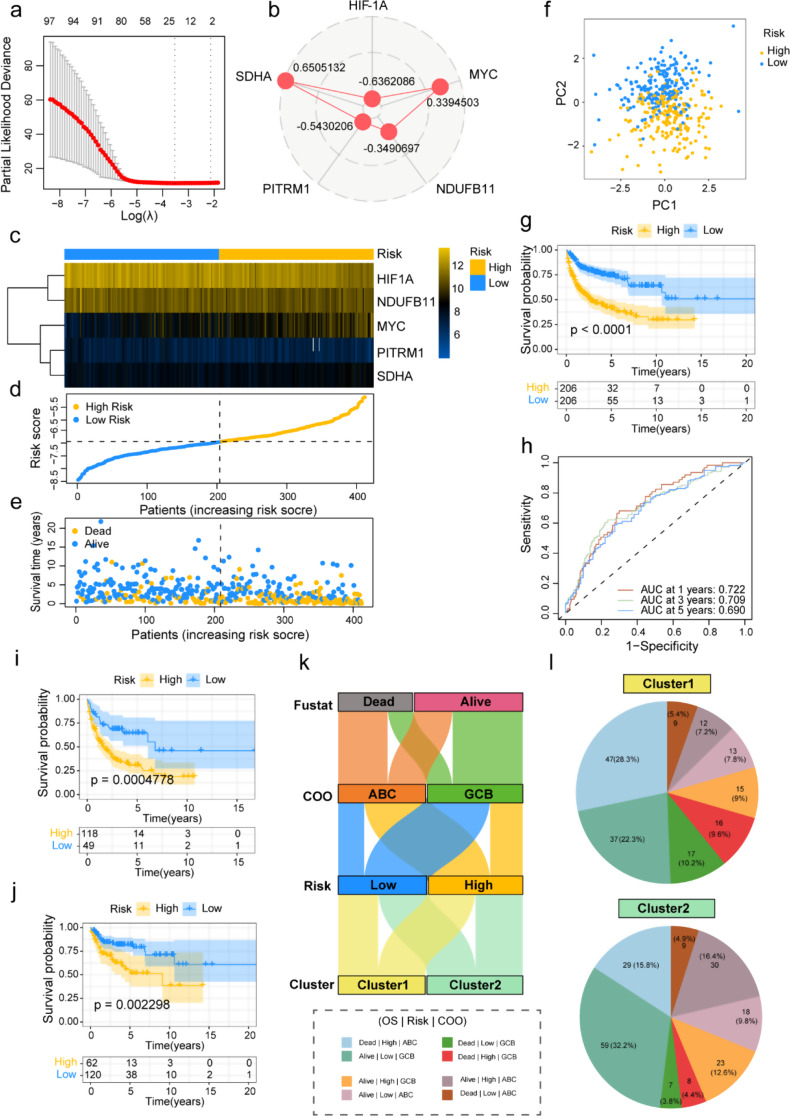
Construction of risk prognosis model based on 5 LMRGs in the training group. **a** LASSO regression model construction. **b** Radar chart displaying the multivariate regression coefficients of five LMRGs. **c-e** The heatmap for the expressions of the five LMRGs, risk score distribution, and survival status in GSE10846. **f** PCA analysis of DLBCL patients in GSE10846. **g** Survival analysis between high and low-risk groups. **h** ROC curve evaluated the predictive performance of models for 1-, 3-, and 5-year. **i**,** j** OS analyses between high- and low-risk groups in DLBCL patients possessed ABC-type (**i**) and GCB-type (**j**). **k** Alluvial diagram of an association between subtypes, risk signature, cell-of-origin (COO) status and survival status. **l** The pie charts representing cluster 1 and cluster 2 in the GSE10846 dataset

Next, we considered selecting representative LMRGs from the aforementioned prognostic-related genes to construct a lactate metabolism-associated model. In total, 98 LMRGs selected were included in the LASSO regression analysis. After determining an optional λ value (Fig. 3a), the non-significant genes were compressed, leaving 24 genes retained. Further, through sequential univariate Cox regression and multivariate Cox regression analyses, 5 LMRGs (HIF-1 A, MYC, NDUFB11, PITRM1, and SDHA) were identified as closely correlated prognostic signatures. Utilizing these 5 LMRGs, we ultimately developed a model associated with the prognosis of DLBCL patients. Moreover, we obtained corresponding coefficients of 5 LMRGs through multivariate Cox regression (Fig. 3b). Each DLBCL patient’s risk score has been calculated by the following formula: risk score = (-0.6362086) × HIF-1 A + 0.3394503 × MYC+ (-0.3490697) × NDUFB11+ (-0.5430206) × PITRM1 + 0.6505132 × SDHA. Based on the median risk score in the training cohort, patients were stratified into high-risk (*n* = 206) and low-risk (*n* = 206) groups, with distinct expression patterns of these five genes across risk groups illustrated in Figure S2a, b, which highlights their differential expression profiles (*p* < 0.001) and median values.

Then, we first analyzed the characteristics through the distribution of the 5 LMRGs, consisting of risk score distribution and survival status between the high-risk and low-risk groups (Fig. 3c, d). As the risk score increases, this subgroup of patients within the high-risk group was more likely to experience more early death than those patients in the low-risk group (Fig. 3e). The PCA revealed a notable distribution of patients into high- and low-risk groups displaying two distinct trends (Fig. 3f). In contrast to the low-risk group, Kaplan-Meier curves showed that the high-risk group had the worse prognosis with shorter OS (*p* < 0.001; Fig. 3g). Besides, the time-dependent ROC analysis assessed the predictive performance of this prognosis-associated risk model, resulting in an AUC of 0.722 at one year, 0.709 at three years, and 0.690 at five years (Fig. 3h). In terms of the origin of cells of the DLBCL, we selected 167 activated B-cell-like (ABC)-subtype DLBCL patients and 182 germinal center B-cell-like (GCB)-subtype DLBCL patients in the training group further to validate the predictive ability of the risk prognosis model. The survival analysis indicated that ABC-type DLBCL patients had worse outcomes than GCB-type DLBCL patients (*p* < 0.001), and ABC-type DLBCL patients had higher risk scores compared to GCB-type DLBCL patients (*p* < 0.001; Fig. S2c, d). Additionally, regardless of the ABC or GCB subtype, the low-risk group had a better prognosis with more prolonged OS than the high-risk group (ABC: *p* < 0.001; GCB: *p* = 0.002; Fig. 3i, j).

Based on the displayed flows in the Sankey diagram, it became evident that high-risk patients, primarily of the ABC subtype from cluster 1, experienced dismal outcomes. Conversely, low-risk patients derived from cluster 2, mainly of the GCB subtype, had a better prognosis (Fig. 3k). Further, we found that in the training group, nearly half of DLBCL patients in Cluster 1 had the ABC subtype, high-risk status, and fatal outcomes, while over half in Cluster 2 had the GCB subtype, low-risk status, and survival outcomes (Fig. 3l). Therefore, multiple analyses of the LMRGs risk prognosis model verified its prognostic prediction efficiency in the training group, including subgroup analysis.

### Validation of the LMRGs‑based risk prognosis model

**Fig. 4:**
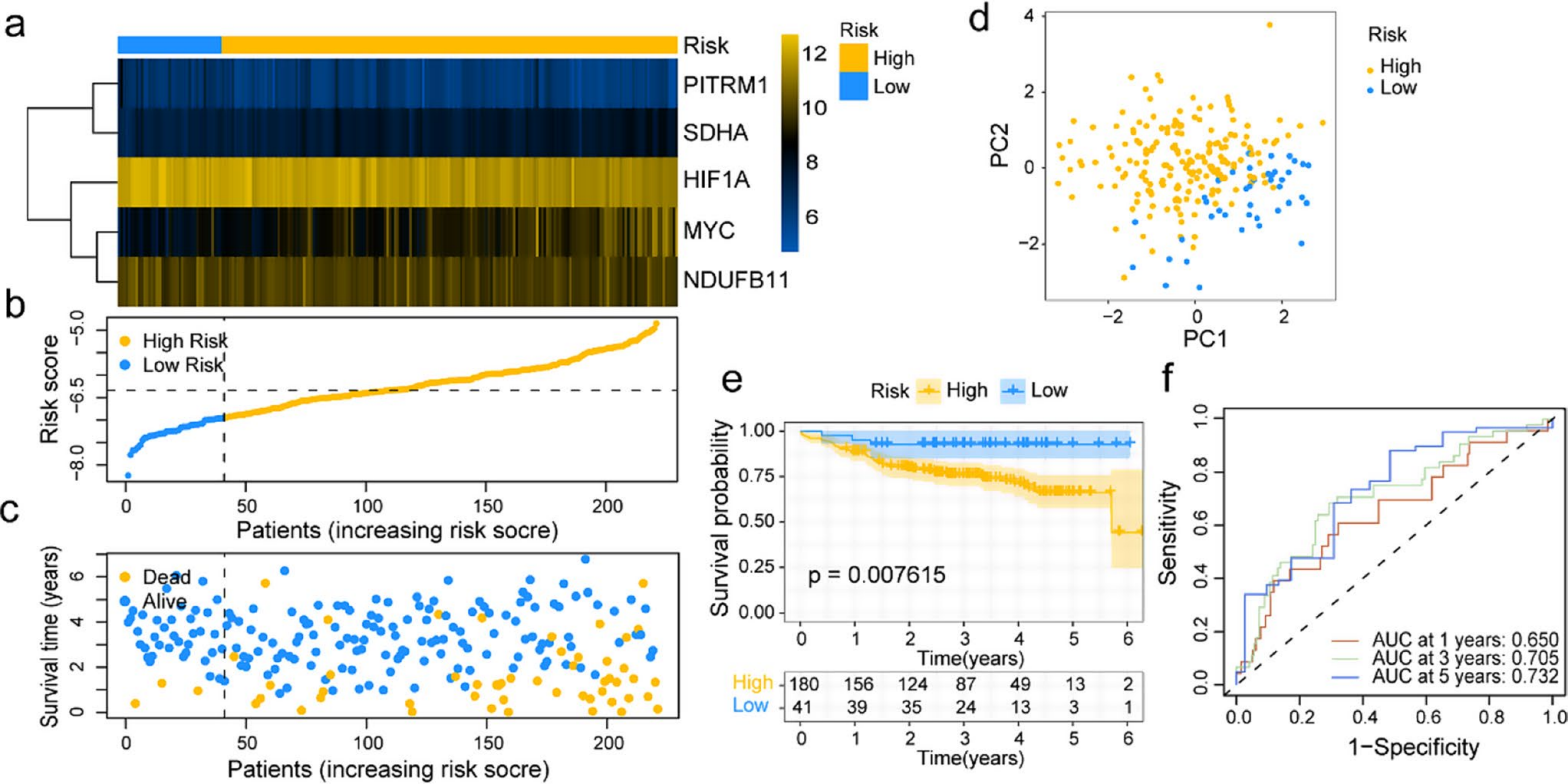
Validation of LMRGs‑related risk prognosis model in GSE87371. **a-c** The heatmap for the expressions of the five LMRGs, risk score distribution, and survival status. **d** PCA analysis of DLBCL patients. **e** Log-rank curves between high and low-risk groups. **f** ROC curves assessed the predictive performances of models for 1-, 3-, and 5-year

The efficacy of the LMRGs‑based risk prognosis model for predicting the prognostic status of DLBCL patients was further confirmed in the validation group. We also set the median risk score of the training group as a cut-off value in the validation group. Figure S3a, b illustrated the expression levels of five key genes in high- and low-risk groups, alongside their median expression values (*p* < 0.001, Fig. S3a, b). Consistent with the above analysis, each patient’s risk score and responding expression profile of 5 LMRGs were shown in Fig. 4a-c. Still, DLBCL patients with higher risk scores tended to have higher expression levels of MYC and SDHA, as well as significantly higher mortality, whereas opposed results in patients with low-risk scores. Furthermore, two distinct areal distributions were pictured according to the risk signature in PCA (Fig. 4d). The survival analysis also indicated that DLBCL patients within the high-risk group presented worse outcomes than those in the low-risk group (*p* = 0.008, Fig. 4e).

Moreover, the AUC of 1 year, three years, and five years were 0.650, 0.705, and 0.732, respectively (Fig. 4f). In subgroup analysis, DLBCL patients belonging to the ABC-type had poorer outcomes with higher risk scores than patients in the GCB-type group (*p* = 0.005) with lower risk scores (*p* < 0.001, Fig. S3c, d). However, there were no statistical differences in survival between these risk signature groups in the ABC-type and GCB-type subgroups (Fig. S3e, f). These findings revealed that the LMRGs-based risk prognosis model can distinguish DLBCL patients with significantly disparate prognoses.

### Correlation analysis of LMRGs-based risk prognosis model with clinicopathological features and independent prognosis analysis

**Fig. 5:**
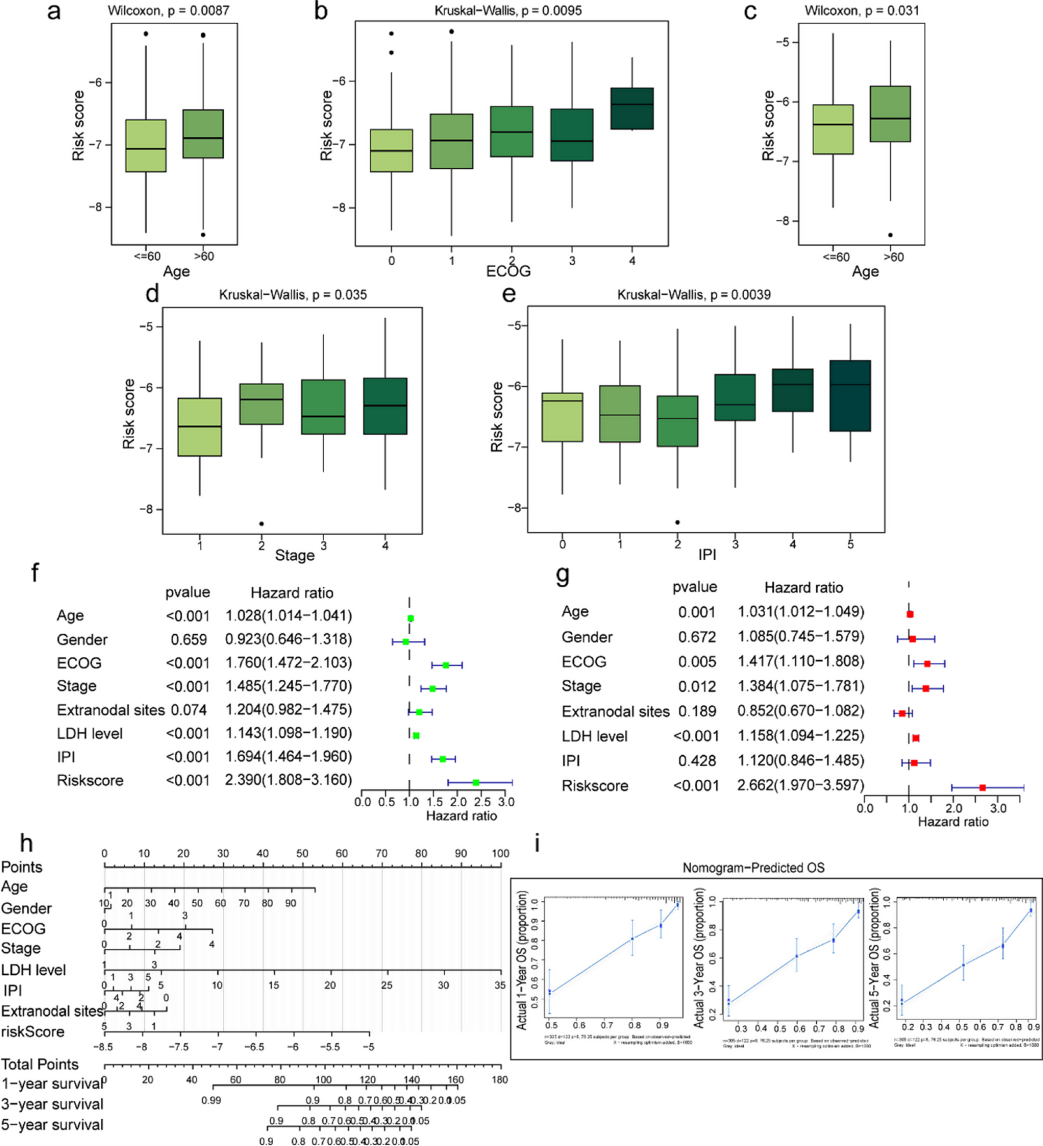
Analyzing clinical associations and independent prognostic analysis of risk scores. **a**,** b** Correlations between risk score and clinical features (age and ECOG PS) in the training group. **c-e** Correlations between risk score and clinical features (age, stage and IPI) in the validation group. **f** Univariate Cox regression analysis of age, gender, ECOG PS, stage, number of extranodal sites, LDH level, IPI and risk score. **g** Multivariate Cox regression analysis of age, gender, ECOG PS, stage, number of extranodal sites, LDH level, IPI and risk score. **h** Development of a nomogram containing eight parameters used in Cox regression analysis. **i** Calibration curves for the nomogram at 1-, 3-, and 5-year

The overall distributions of data with clinicopathologic variables and risk signatures in both the training and validation groups are presented in Figure S4a, b. Additionally, the correlation between risk stratification and clinicopathological features was analyzed in both the training and validation groups, as detailed in Table S3. First, we conducted the correlation analysis of the risk scores with pathological features using the Mann-Whitney U or Kruskal-Wallis sum test. Among these seven variates, including age, gender, Ann Arbor stage, IPI, lactate dehydrogenase (LDH) level, number of extranodal sites, and ECOG PS, the results suggested that a higher risk score was associated with older age and higher ECOG PS (*p* < 0.01, Fig. 5a, b). However, no significant differences were observed by gender, LDH level, stage, or number of extranodal sites in the training group (Fig. S4c-g). However, the abnormal LDH level subgroup exhibited a relatively higher risk score than the normal LDH level subgroup, yet statistical significance has not been achieved. In the validation group, we also observed risk scores correlated with age, stage, and IPI (*p* < 0.05, Fig. 5c-e), except for gender (Fig. S4h). These findings denoted that the risk score calculated based on the risk prognosis model had the potential for clinical stratification.

Next, we employed univariate and multivariate Cox regression analyses to assess the risk model as an independent prognostic factor for determining patients’ prognosis in the training group (Fig. 5f, g). In addition, a nomogram that integrated risk score and seven clinicopathologic parameters was constructed to predict outcomes for DLBCL patients quantitatively. The nomogram showed that the total point of each patient measured by all parameters, especially risk score, occupied the predominant proportion. It also provided the corresponding survival probability at 1-, 3-, and 5-year (Fig. 5h). The calibration curve was used to validate the satisfactory performance of the LMRGs-based risk prognosis model, which showed good consistency between predicted 1-, 3- and 5-year OS and actual OS (Fig. 5i). Therefore, the results mentioned above demonstrated the strong feasibility and practicality of our prognosis model in clinical settings.

### Correlation analysis of response to immunotherapy and immune landscape in different risk groups

**Fig. 6:**
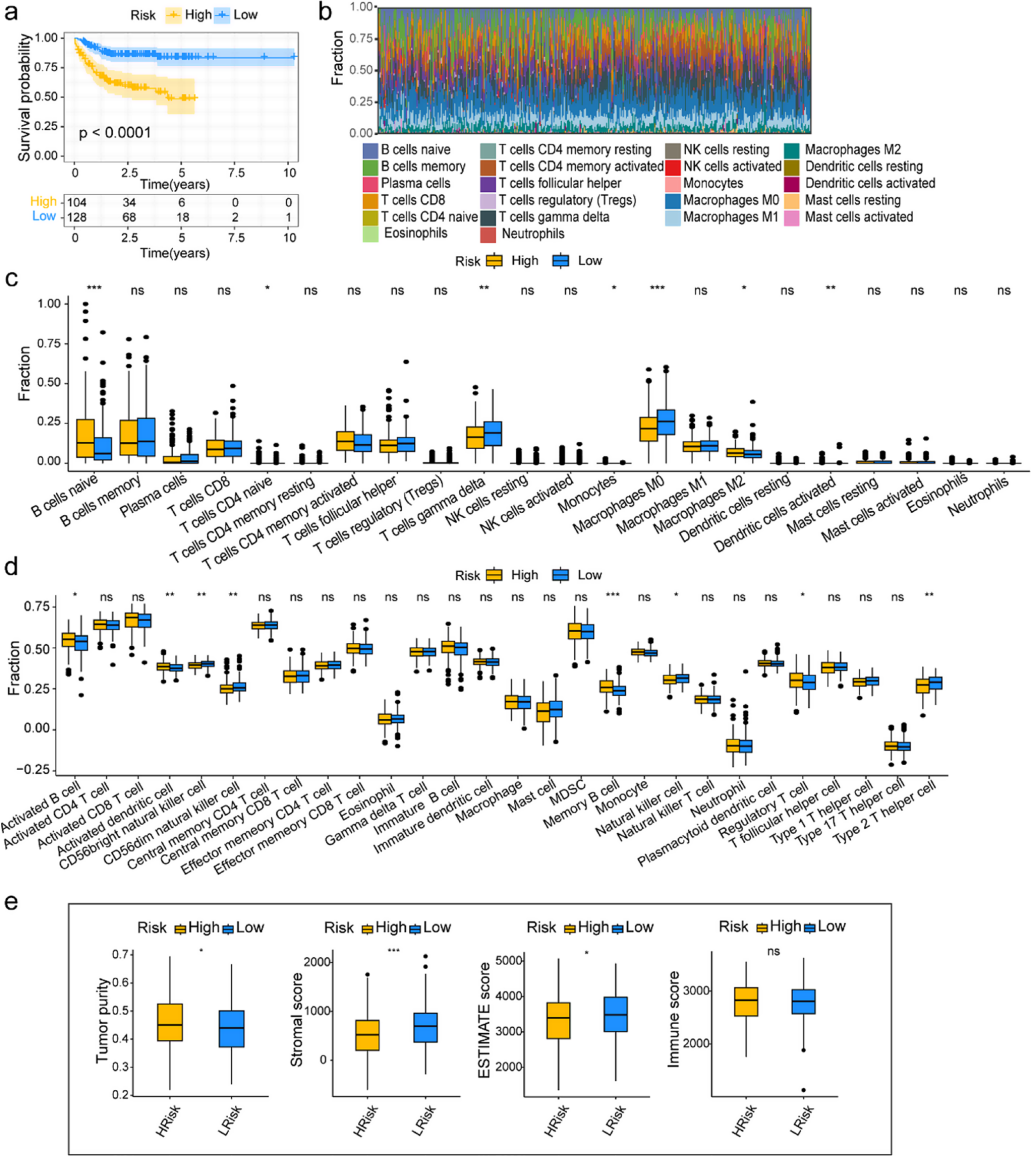
The different immune status between the low- and high-risk groups in the training dataset. **a** Survival analysis between high and low-risk groups in DLBCL patients received R-CHOP (rituximab plus cyclophosphamide, doxorubicin, vincristine, and prednisone) treatment. **b** The CIBERSORT algorithm was employed to assess the immune cell proportions. **c-e** The immune profiles of high- and low-risk groups were analyzed using the CIBERSORT method (**c**), the ssGSEA method (**d**), and the ESTIMATE method (**e**)

Further, we identified the prognosis efficacy of the risk prognosis model in DLBCL patients with immunochemotherapy treatment, and the survival curve indicated the patients in the high-risk group tended to experience shorter survival duration and poorer outcomes than the low-risk group (*p* < 0.001, Fig. 6a). The risk model’s accuracy in predicting patient outcomes may be contingent on the associations with different immune TME compositions, especially the immune and stromal component abundance. First, we created a bar chart illustrating the distribution of 22 cell types in each patient using the CIBERSORT method (Fig. 6b). As shown in Fig. 6c, compared to the low-risk group, B cell naive cells and macrophages M2 had higher proportions in the high-risk group (*p* < 0.05), whereas T cells gamma delta and macrophages M0 had lower proportions (*p* < 0.01). Moreover, the ssGSEA method incorporated 28 types of immune cells to re-evaluate the immune cell infiltration in the TME. Similarly, we found the patients within the high-risk group tended to obtain the activated B cells, activated dendritic cells, memory B cells, and Treg cells but have lower proportions of CD56 bright NK cells, CD56 dim NK cells, NK cells and type 2 T helper cells than those in the low-risk group (*p* < 0.05, Fig. 6d).

We also analyzed the correlations between risk scores and various immune cells, and the results showed that two types of immune infiltrating cells were positively associated with risk scores: activated CD8 T cells and macrophages. Gamma delta T cells, central memory CD8 T cells, central memory CD4 T cells, T follicular helper cells, effector memory CD4 T cells, type 1 T helper cells, eosinophils, NK cells, mast cells, type 2 T helper cells, CD56 dim NK cells, and CD56 bright NK cells were significantly negatively correlated with the risk score (Fig. S5a). Moreover, the high-risk group exhibited higher tumor purity but lower stromal and ESTIMATE scores compared to the low-risk group (*p* < 0.05, Fig. 6e). We also focused on the relationships between risk score and immune checkpoint targets in DLBCL. The results suggested that three out of eight immune checkpoint molecules, including SIRPA (SIRPα), PDCD1 (Programmed death-1; PD-1), and CD47, exhibited upregulated expression in the high-risk group compared to the low-risk group (*p* < 0.01, Fig. S5b). Based on multiple immune analyses, the correlation between the immune landscape and LMRGs-related risk score highlighted the risk model’s significant value in assessing the immune microenvironment.

### Correlation analysis of LMRGs-based risk prognosis model with drug sensitivity and therapeutic response, and enrichment analysis of DEGs between two risk groups

**Fig. 7:**
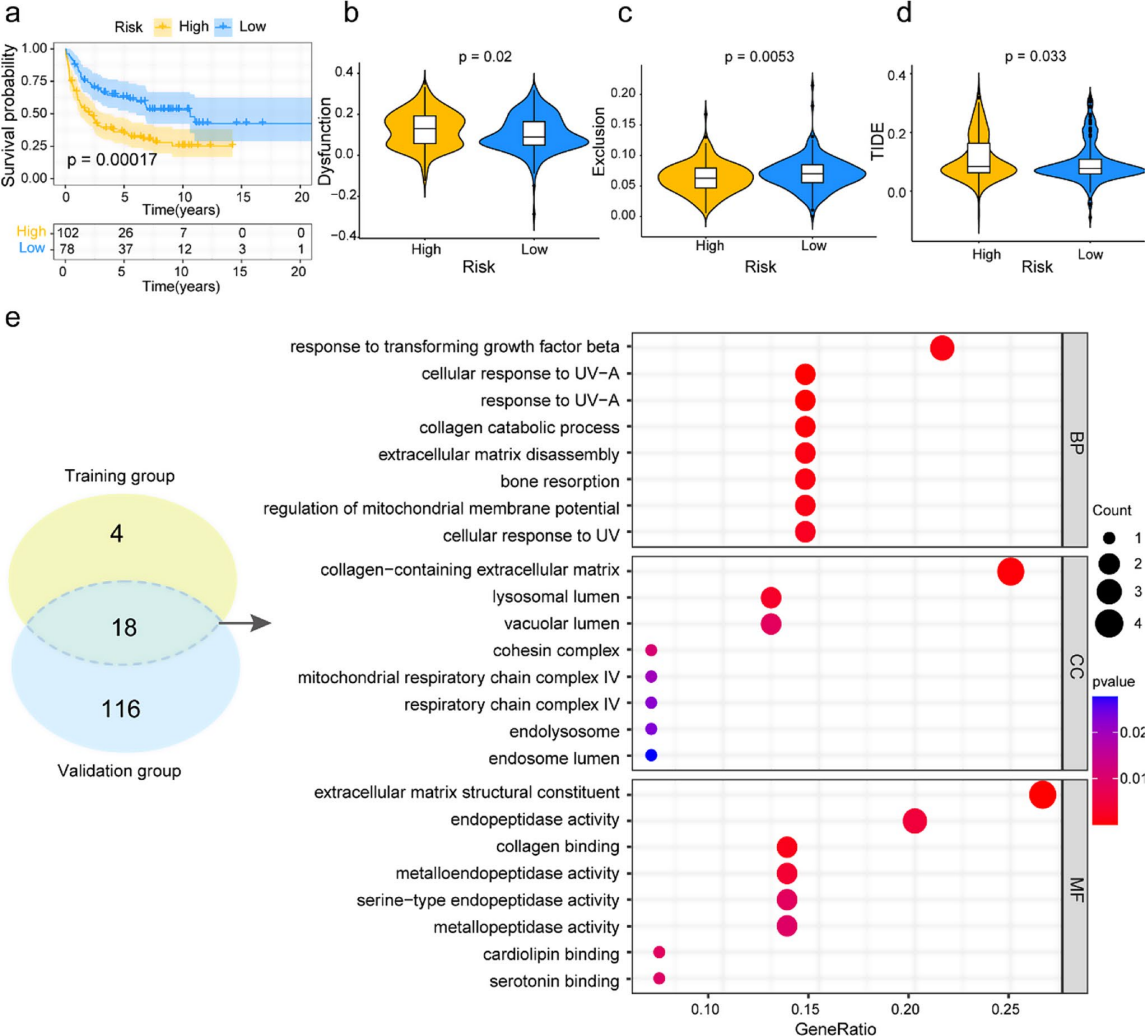
Analyzing drug sensitivity, therapeutic response, and functional enrichment in high- versus low-risk groups. **a** The OS analysis between high- and low-risk groups with DLBCL patients received CHOP treatment. **b-d** TIDE analysis. **e** GO analysis was performed on the common set of differentially expressed genes between the high-risk and low-risk groups selected from the GSE10846 and GSE87371 datasets

In addition to immunochemotherapy treatment (R-CHOP), the LMRGs-based risk prognosis model exhibited robust predictive efficacy in traditional chemotherapy (CHOP: cyclophosphamide, doxorubicin, vincristine, and prednisone). Comparison of OS between high-risk and low-risk groups in the training group showed significant prognostic disparity (*p* < 0.001, Fig. 7a). To explore the potential relationship between the risk score and drug sensitivity, IC50 values were estimated for nine medications commonly used in clinical treatment or trials for DLBCL. As presented in Figure S6a-k, cisplatin, cytarabine, bortezomib, ruxolitinib, temozolomide, vinblastine, and vinorelbine possessed more outstanding sensitivity in the high-risk group with lower IC50 value than in the low-risk group (*p* < 0.01), whereas ibrutinib, rapamycin, venetoclax and gemcitabine had no statistical significance in therapeutic efficacy. Furthermore, TIDE is a computational method to assess tumor immune function and immune exclusion. The result of the TIDE analysis showed the high-risk group characterized by a higher dysfunction score, a lower exclusion score, and a higher TIDE score (*p* < 0.05, Fig. 7b-d), which demonstrated these patients were more likely to respond to immune checkpoint inhibitors, while the blunt response in the low-risk group.

Next, to investigate the potential biological pathways correlated with LMRGs signature, we used the GO analysis according to the DEGs between the high-risk and low-risk groups, which screened 22 DEGs and 134 DEGs in the training and validation groups, respectively. Then, 18 DEGs with consistent expression abnormalities were merged into a new gene set to conduct GO enrichment analysis via the clusterProfiler R package. The discrepancies between the high-risk and low-risk groups might be attributed to differences in BP, CC, and MF among these DEGs. Genes enriched in BP were mainly related to the response to the transforming growth factor beta/ultraviolet-A (UV-A)/UV, collagen catabolic process, extracellular matrix disassembly, and regulation of mitochondrial membrane potential. Genes enriched in CC were involved in collagen-containing extracellular matrix, lysosomal/vacuolar/endosome lumen, cohesion complex, mitochondrial respiratory chain complex IV, and endolysosome. Genes enriched in MF were predominantly associated with extracellular matrix structural constituent, peptidase activity, and collagen/cardiolipin/serotonin binding (Fig. 7e).

Therefore, these findings exhibited that patients belonging to the high-risk group may benefit from immune checkpoint inhibitors, and the potential biological pathways based on the differences between the high-risk and low-risk groups suggested a possible therapeutic entry point.

### Cell-cell communication analysis among immune cells based on the LMRGs risk scores

**Fig. 8:**
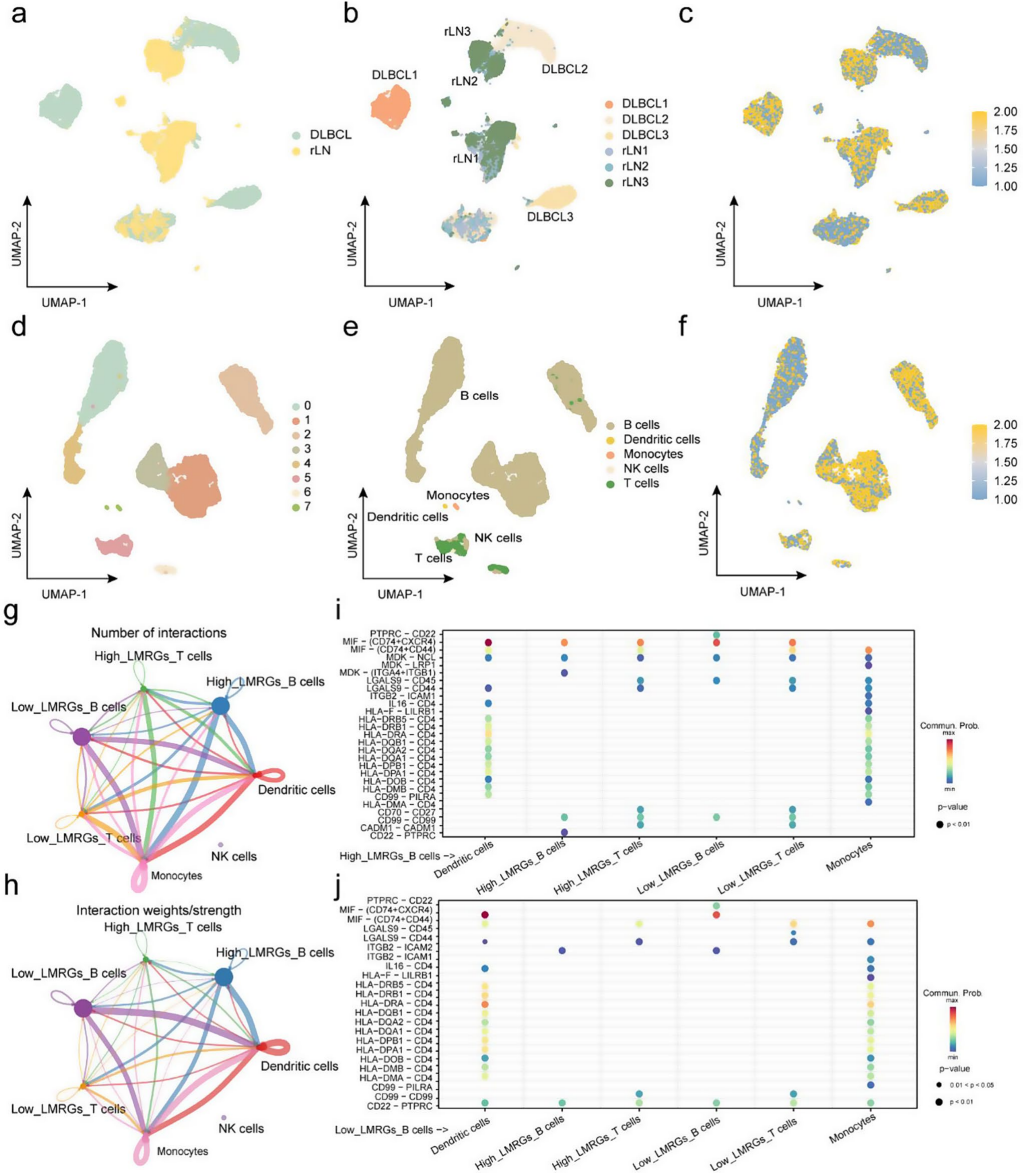
Analysis of cell-cell communication. **a-b** UMAP plots of reactive lymphoid tissue and DLBCL. **c** UMAP plot of LMRGs risk scores for all cells included in the analysis. **d** Dimensionality reduction and clustering of single-cell sequencing data from DLBCL tissue identified 7 distinct clusters. **e** Clusters were annotated based on marker genes of immune cells. **f** UMAP plot displaying LMRGs risk scores across different immune cells. **g-h** The integrated cell-cell communication network illustrated the number of interactions and the weights of interactions between distinct immune cell clusters. **i-j** Bubble plots showed receptor-ligand pairs between high and low LMRGs risk score B cells and other cells

To further understand the cell-cell communication in case with high or low LMRGs signature, we leveraged scRNA-seq datasets containing three non-malignant and three malignant lymph node samples. Figure 8a and b depicted the cells of 6 samples mapped onto a Uniform Manifold Approximation and Projection (UMAP) plot, and each LMRGs risk score was subsequently calculated based on the formula above by integrating the expression of the 5 genes (Fig. 8c). Then, how such cell interactions were involved in the DLBCL microenvironment among immune cells with different LMRGs risk scores has been further analyzed (Fig. 8d). Three lymph node samples of DLBCL consisted of major B cells and T cells as well as minor NK cells, monocytes and dendritic cells (Fig. 8e). Further, the performance of LMRGs risk scores within these 5 cell types was visualized in Fig. 8f, and B cells and T cells could be classified into high LMRGs signature group and low LMRGs signature group, respectively. The circle plots indicated the number and weights of cellular communication between different myeloid cells and lymphocytes (Fig. 8g, h). Based on the prediction and analysis of cell communication networks, B cells with high LMRGs signature engaged with T cells through the MIF–(CD74 + CXCR4) pathway, compared to the low LMRGs signature group. Furthermore, B cells in the low LMRGs signature group interacted with T cell, dendritic cells and monocytes via the CD22-PTPRC pathway. In addition, B and T cells exhibiting low LMRGs signature could interact through the LGALS9-CD45 pathway (Fig. 8i, j).

### High expression of SDHA is indicative of poor prognosis and promotes proliferation

**Fig. 9:**
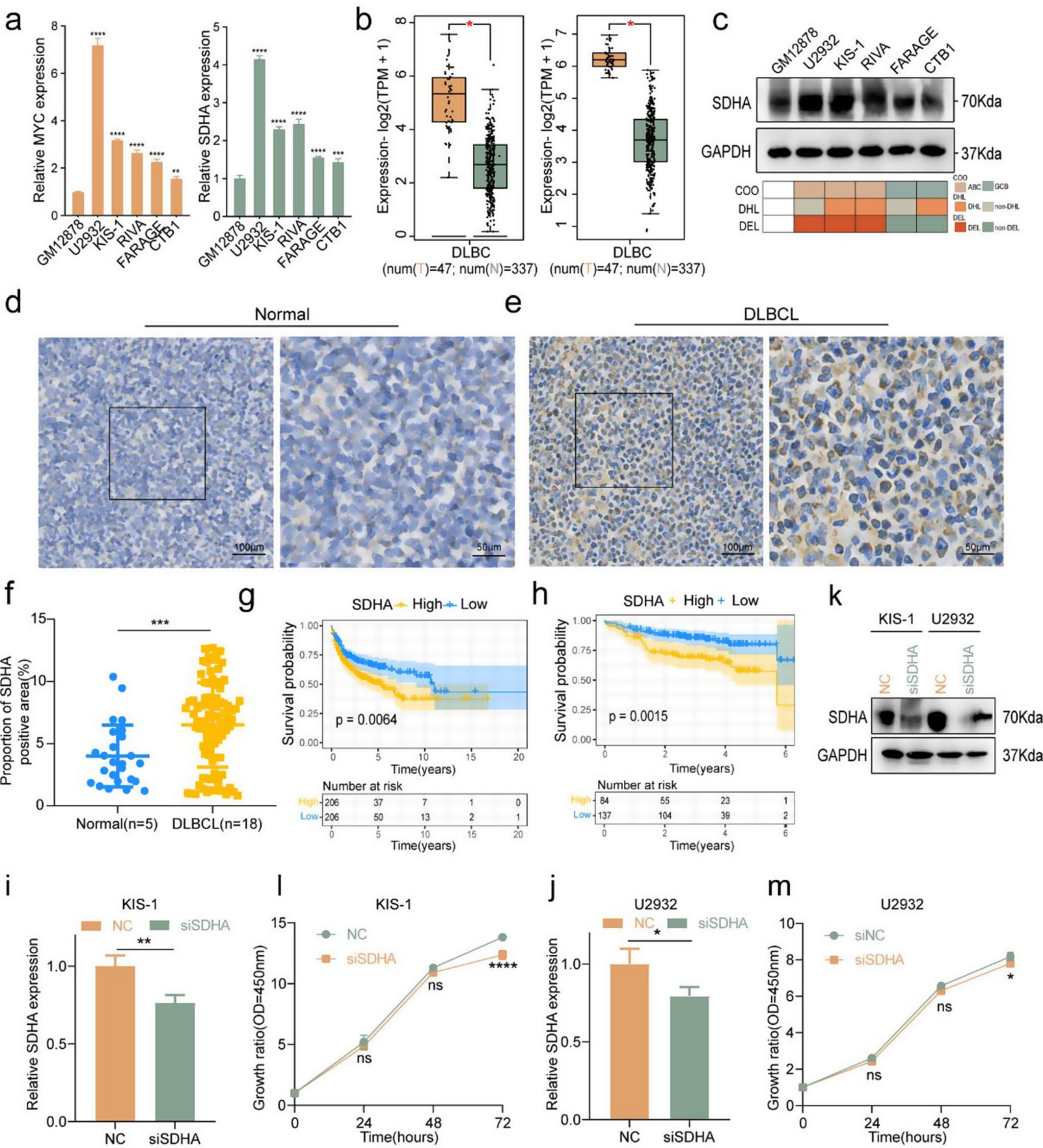
The high expression of SDHA promotes DLBCL proliferation. **a** The RNA expression levels of MYC and SDHA in normal B cell line and various DLBCL cell lines. **b** The RNA expression levels of MYC (left) and SDHA (right) in normal and DLBCL tissues from the GEPIA database. **c** The protein expression levels of SDHA were measured in a normal B cell line and various DLBCL cell lines. **d-f** Immunohistochemical analysis of SDHA protein levels in DLBCL tissues compared to normal tissues. **g-h** High SDHA expression is associated with poor survival in the GSE10846 and GSE87371 datasets. **i-m** QRT-PCR and Western blot were used to assess the changes in the RNA and protein expression levels of SDHA after siRNA treatment in two DLBCL cell lines (i-k). CCK-8 assay demonstrated that cell proliferation was significantly inhibited 72 h after siRNA treatment (l-m). Abbreviations: DHL: double-hit lymphoma; DEL: double-expressor lymphoma

To investigate the expression of genes with positive coefficients in DLBCL, we examined the transcriptional levels of *MYC* and *SDHA* in a normal B cell line and in DLBCL cell lines, as well as in samples from normal individuals and DLBCL patients. The results of qRT-PCR revealed that the expression of *MYC* and *SDHA* was elevated in DLBCL cell lines compared to a normal B cell line (*p* < 0.01, Fig. 9a). Moreover, in DLBCL patient samples, *MYC* and *SDHA* expression levels were consistently higher than those in normal controls (*p* < 0.05, Fig. 9b). Given that MYC is an established adverse prognostic factor [23], this study focused on the expression level and role of SDHA in DLBCL. The Western blot results confirmed that SDHA protein levels were higher in most DLBCL cell lines compared to a normal B cell line (Fig. 9c), and IHC results similarly showed that SDHA expression was elevated in DLBCL patient tissues compared to normal tissues (*p* < 0.001, Fig. 9d-f). Survival analysis indicated that high *SDHA* expression predicts poor prognosis in DLBCL (*p* < 0.01, Fig. 9g-h). To further investigate the functional role of SDHA in DLBCL pathogenesis, We employed siRNA-mediated knockdown of SDHA in two DLBCL cell lines and observed a reduction in RNA levels at 48 h post-transfection, followed by a decrease in protein levels at 72 h post-transfection. (*p* < 0.05, Fig. 9i-k). Additionally, the CCK-8 assay demonstrated that decreased SDHA expression resulted in suppressed cell proliferation at 72 h (*p* < 0.05, Fig. 9l-m). These findings suggested that high SDHA expression may drive cell proliferation and is linked to unfavorable prognosis in DLBCL patients.

## Discussion

Over the past two decades, advancements in understanding DLBCL regarding molecular classification, biological diversity, and prognostic markers have contributed to predicting the OS of DLBCL patients. However, beyond the conventional IPI, there is a need for tools integrating subtype identification and outcome stratification. Multi-gene approaches, more accurate than single-gene analyses, hold significant potential.

Cancer cells exhibit metabolic plasticity, adapting to microenvironmental fluctuations through processes like oxidative glycolysis and lactate production [24, 25]. This metabolic reprogramming, involving enhanced glucose uptake, glycolytic enzyme activity, and lactate transporters, underscores the Warburg effect’s role in carcinogenesis [26]. The discovery of lactagenesis for carcinogenesis has elevated the existing significance of the Warburg effect and provided potential therapeutic breakthroughs. In this study, we investigated LMRGs in DLBCL. Univariate Cox regression identified 98 prognosis-associated LMRGs, stratifying patients into two subtypes: cluster 1 and cluster 2. Cluster 1 exhibited shorter OS, increased Tregs and T follicular helper cells, and reduced immune cell infiltration, suggesting an immunosuppressive microenvironment. This cluster also showed elevated expression of immune checkpoint genes, including *CD274* (PD-L1), *CTLA4*, *HAVCR2* (TIM-3), *LGALS9* (Galectin-9), *CSF1R*, *TGFB1*, and *CD244*. PD-L1 overexpression induces T-cell exhaustion via PD-1/PD-L1 [26], while anti-PD-1/anti-CD20 therapy enhances T-cell activity in DLBCL models [27]. CTLA-4 blockade with ipilimumab elicits durable responses in select DLBCL cases [28], though broader validation is needed. The TIM-3/Galectin-9 axis impairs CD8 + tumor-infiltrating lymphocytes (TIL) function and correlates with chemotherapy resistance [29]. CSF1R, paradoxically associated with favorable prognosis in DLBCL, remains a therapeutic target for reprogramming M2-tumor-associated macrophages (TAMs), as shown by SYHA1813’s (a VEGFR and CSF1R inhibitor) efficacy against Bruton’s tyrosine kinase inhibitor (BTKi) resistance [30]. TGFB1 displays a dual role, promoting pro-tumorigenic networks yet predicting enhanced immune checkpoint blockade (ICB) responses [31]. CD244, a signaling lymphocyte activation molecule (SLAM) family member, may confer tumor-protective effects [32]. The findings demonstrated that the TME composition and immune checkpoint expression profiles in cluster 1 served as key determinants of patient prognosis and critical therapeutic entry points for overcoming drug resistance mechanisms. Furthermore, Cluster 1 exhibited upregulated glucose metabolism activity and a heightened cellular response to varying oxygen levels, suggesting an adaptive approach to enhanced nutrient uptake and rapid growth.

Next, five LMRGs with significant prognosis values were screened in the DLBCL cohort via COX regression and LASSO estimation, including *HIF-1 A*, *MYC*, *SDHA*, *NDUFB11*, and *PITRM1*. Among these key genes, *MYC* and *SDHA* acted as risk factors, i.e., increased expressions of *MYC* and *SDHA* in DLBCL patients had poor outcomes, while other key genes were protective factors in DLBCL patients. *MYC*, a widely studied central gene, was major in deriving human tumorigenesis. Except for regulating the expression of specific target genes, the oncogenic mechanism is likely to modulate the transcription machinery [33]. The performance of *MYC* biology supports bioenergetic and biosynthetic activities, particularly in abnormal metabolism, throughout the entire process of tumor development. For example, *MYC* can act as a regulator of glycolysis, influencing glucose transport and conversion into lactate by targeting specific genes, thereby promoting tumorigenesis [34]. Succinate dehydrogenase complex subunit A (SDHA) belongs to mitochondrial respiratory chain complex II and is involved in electron transfer in the respiratory chain and succinate catabolism in the Krebs cycle [35]. Similarly, MYC-mediated SDHA acetylation plays a crucial role in tumor cell growth via epigenetic regulation [36]. According to the previous reporter, SDHA was upregulated in non-small-cell lung cancer with metabolic disorders [37], which were also found in highly metastatic uveal melanoma [38] and clear cell renal cell carcinoma [39].

Based on the five LMRGs, we constructed a risk prognosis model with good performance for DLBCL patients in the training and validation groups. As an independent risk factor, the risk model was used to categorize the DLBCL population into two risk groups and effectively predicted the outcome of the DLBCL cohort with AUC > 0.7 in the training cohort. However, we observed slightly superior predictive performance in the training set compared to the validation group. This discrepancy may be attributed to potential differences in sample sources, data collection timeframes, or batch effects arising from variations in experimental techniques. Additionally, the limited sample size in the validation cohort could have contributed to this divergence. Furthermore, other molecular subtypes based on whole-exome sequencing, beyond COO classification, might influence the model’s performance, although we currently lack such data for comprehensive analysis. These factors highlight the need for larger, more diverse cohorts and additional molecular profiling to further refine and validate the model’s generalizability.

Considering that the risk score represented lactate metabolism-related features, we comprehensively depicted the LMRGs signature closely associated with prognosis status, immune infiltration, immune checkpoint molecule expression, and drug sensitivity. Additionally, our model could be applied to DLBCL subgroups based on COO classification, which was identified by gene expression profiling [2], indicating that the new LMRGs signature exhibited strong predictive capabilities in both ABC and GCB subgroups, further suggesting that lactate metabolism may impact tumor progression.

According to the immune infiltration degree analysis between the high- and low-risk group, the anti-inflammatory cells, such as Macrophages 2 and Tregs, presented higher levels in the high-risk group than those in the low-risk group. Moreover, given the high-risk group exhibited more pronounced characteristics related to lactate metabolism than the low-risk group, we proposed that the poor prognosis in the high-risk group may be linked to an adverse immune landscape caused by lactate metabolism. Lactate metabolism drives tumorigenesis by increasing immunosuppressive cells in the microenvironment, thereby creating an immunosuppressive milieu [40]. Macrophages, which are widely distributed across all tissues, are highly plastic cells with significant functional diversity. Commonly, activated macrophages are divided into two subtypes dependent on the polarization state: M1 macrophages are characterized by the pro-inflammatory phenotype and rely on glycolysis for energy, and M2 macrophages are responsible for immune regulation and tissue repair [41]. During tumor malignancy transformation, tumor-associated macrophages, initially displaying pro-inflammatory and antitumor responses, shift towards the M2 phenotype under the induction of tumor-derived lactate [41]. Tregs are a subgroup of T cells that profoundly impact the immune homeostasis in the immune system while displaying a representative pro-cancer composition in TME [11, 42]. Of note, Tregs possess specific traits in metabolic flexibility. Briefly, Tregs, based on the toleration of high lactate, can upregulate the lactate metabolism to avoid the destabilizing condition in high-glucose concentration, whereas the need for lactate uptake is dispensable [11]. Alessia Angelin et al. revealed that these metabolic adaptations in Tregs are a result of the mechanism by which Foxp3 can orchestrate the transcriptional repression of Myc, inhibit glycolysis, and confer upon Tregs the ability to withstand the inhibitory effects of lactate [43]. Therefore, lactate metabolism can integrate various features of the TME, encompassing cellular bioenergetics, biosynthesis, and signaling to bolster tumor progression and prognosis.

Given the less common clinical responses to immune checkpoint inhibition in hematologic tumors, developing dependable predictive biomarkers poses formidable challenges [44]. Here, we assessed the expressions of immune checkpoint targets in DLBCL to reveal that in the case of patients in the high-risk group, PD-1 presented higher expression than the low-risk group. Accordingly, adding anti-PD-1 into the standard treatment may improve the patient’s prognosis within the high-risk group based on the algorithm in our risk prognosis model. However, recent research has shown that lactic acid stimulation enhances the PD-1 expression in Tregs, potentially contributing to the ineffectiveness of PD-1 blockade therapy [45]. Furthermore, the release of lactic acid by cancer cells establishes a connection between the redistribution of TAMs and the regulation of PD-L1/PD-1, ultimately leading to T-cell apoptosis and promoting immune evasion in vitro [46]. Thus, despite the availability of adequate conditions for receiving anti-PD-1 inhibitors, it is necessary to assess the immune-suppressive cell subsets and acidity in the microenvironment to evaluate the reliable response of patients with high-risk scores to immune checkpoint blockade. Unfortunately, we lack extensive patient data to thoroughly analyze our risk prognosis model for immune checkpoints. Indeed, other immune checkpoints, such as CD47 and SIRPA, should also be considered for inclusion in the treatment regimen for the high-risk group due to their high expression. Moreover, through single-cell RNA sequencing (scRNA-seq) technology, we identified potential cell-cell communication pathways between immune cells with different LMRGs risk scores. For example, B cells with a high LMRGs signature interacted with T cells via the MIF–(CD74 + CXCR4) pathway, while those with a low LMRGs signature associated with T cells, dendritic cells, and monocytes through the CD22-PTPRC pathway. These targets in these pathways may be incorporated into standard protocols to enable personalized therapy.

The R-CHOP regimen, which comprises vincristine, remains the optimal choice for DLBCL patients. Our drug sensitivity analysis demonstrated that drugs similar to vincristine, such as vinblastine and vinorelbine, also exhibited notable sensitivity in the high-risk group. In salvage chemotherapy regimens, we assessed the sensitivity of several common drugs, including cisplatin, cytarabine, and gemcitabine. The results suggested that high-risk patients may consider salvage therapy that includes cisplatin and cytarabine, such as the DHAP (dexamethasone, cisplatin, and cytarabine) regimen. Temozolomide and ruxolitinib could be potential candidates for inclusion in the treatment regimens for high-scoring patients due to the observation of higher sensitivity in the high-risk group. TIDE analysis indicates a favorable response to immunotherapy in the high-risk group. Therefore, high-risk group patients are highly likely to achieve disease remission and extended survival when receiving sensitive chemotherapy alongside immunotherapy regimens.

The LMRG-based prognostic risk model, initially developed using Affymetrix gene expression data, demonstrates clinical translatability through real-time quantitative PCR (RT-qPCR) validation. While the Affymetrix platform enables standardized high-throughput transcriptional profiling, RT-qPCR offers superior cost-effectiveness and accessibility for routine clinical implementation. Future clinical adoption of this model will require protocol optimization for RT-qPCR-based detection and rigorous multicenter validation studies.

In addition, we assessed the prospective utility of implementing our risk prognosis model in clinical application. From this perspective, we made a nomogram, incorporating both risk score and clinical pathological parameters, which serves as a visual tool to aid clinicians in prognostic prediction for DLBCL patients. Moreover, the calibration plot demonstrated a strong consistency between the survival probability predicted by the nomogram and the actual survival observed in the real world. To concretely explore the engagement of SDHA in DLBCL, we employed various experimental methods, including qRT-PCR, Western blot, IHC, and CCK-8. The results supported that high expression of SDHA tended to promote DLBCL cell growth. However, the explicit mechanism by which SDHA-driven lactagenesis contributes to carcinogenesis requires further investigation.

Although this study demonstrated the value of the LMRGs-based risk prognosis model constructed from sequencing and clinical data, it had certain limitations. First, results from bioinformatics analysis required validation with our samples for greater reliability. Second, the heterogeneity of database data may have introduced bias, limiting the model’s applicability across DLBCL patient groups. Finally, the SDHA function was validated only with a CCK8 proliferation assay, which may not fully capture its role in DLBCL. Further functional studies are needed to clarify SDHA’s impact on lactate metabolism and disease progression.

## Conclusions

Our study identified two lactate metabolism-related subtypes and employed a LASSO-Cox regression approach to discern and screen for LMRGs with significant prognostic implications. We successfully constructed a risk prognosis model based on five representative LMRGs, which demonstrated reliability in predicting the prognosis and risk stratification of DLBCL patients. Subsequently, multiple analyses were made, including clinical characteristics, immune profile, and drug response based on the risk prognosis model. Our analysis revealed a potential link between lactate metabolism and immune cell distribution within the TME. These findings align with existing evidence suggesting that lactate metabolism may influence the immune landscape, warranting further exploration of its biological mechanisms and possible therapeutic applications in immunotherapy.

## Electronic supplementary material

Below is the link to the electronic supplementary material.

Supplementary Material 1

## Data Availability

No datasets were generated or analysed during the current study.
