# Supplementary material for: Identification of molecular clusters and a risk prognosis model for diffuse large B-cell lymphoma based on lactate metabolism-related genes
Source: Ann Hematol. 2025 Apr 5;104(5):2847–67. doi: 10.1007/s00277-025-06321-1 (PMC12141129; doi:10.1007/s00277-025-06321-1)
Supplement: Supplementary file 1 — Supplementary Material 1 [file 277_2025_6321_MOESM1_ESM.zip › Supplementary File20250225/Original Images for Blots_Gels/Original image files for the blots included in figures.pptx]

## Slide 1
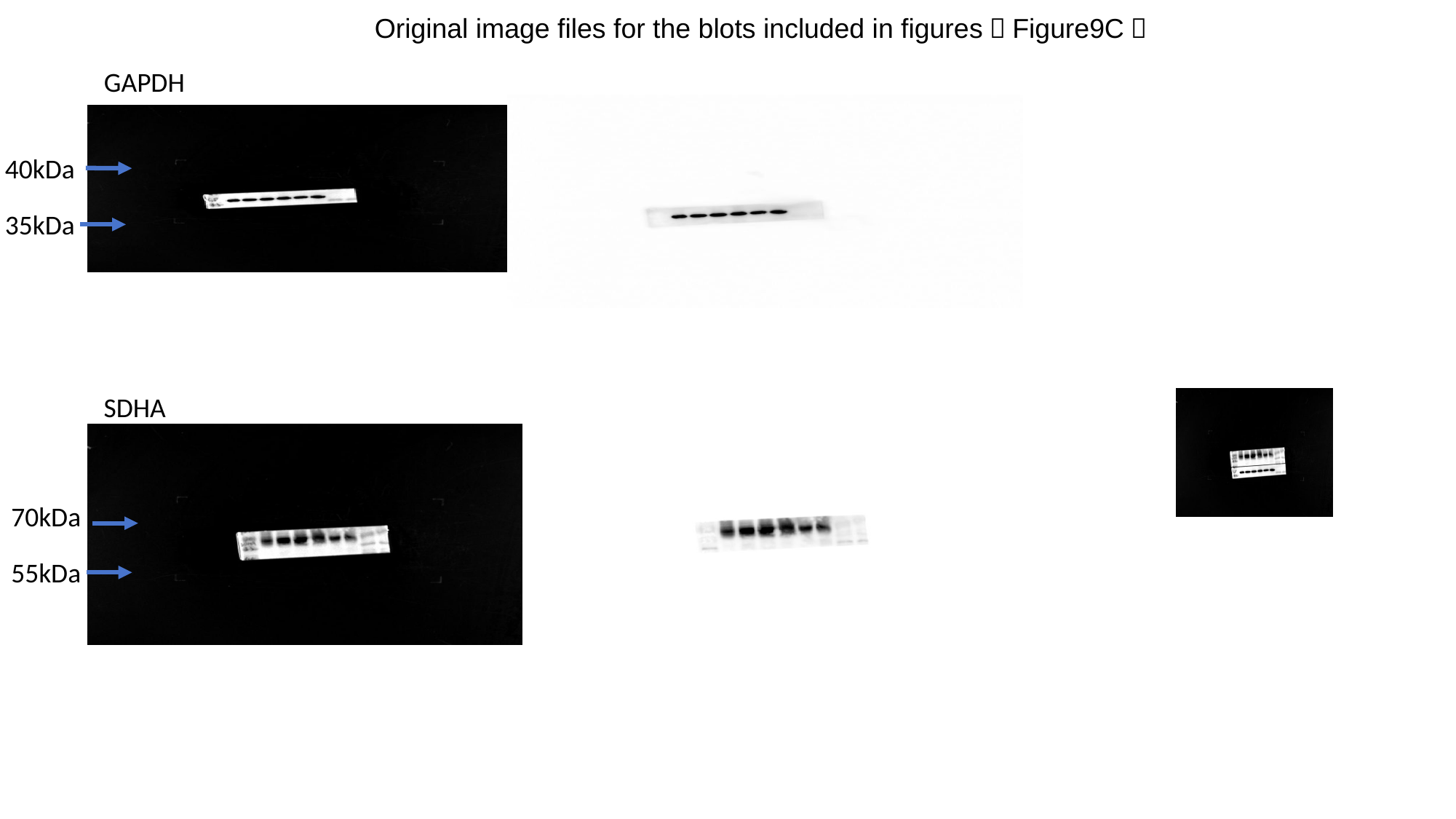

Original image files for the blots included in figures（Figure9C）
GAPDH
40kDa
35kDa
SDHA
70kDa
55kDa

## Slide 2
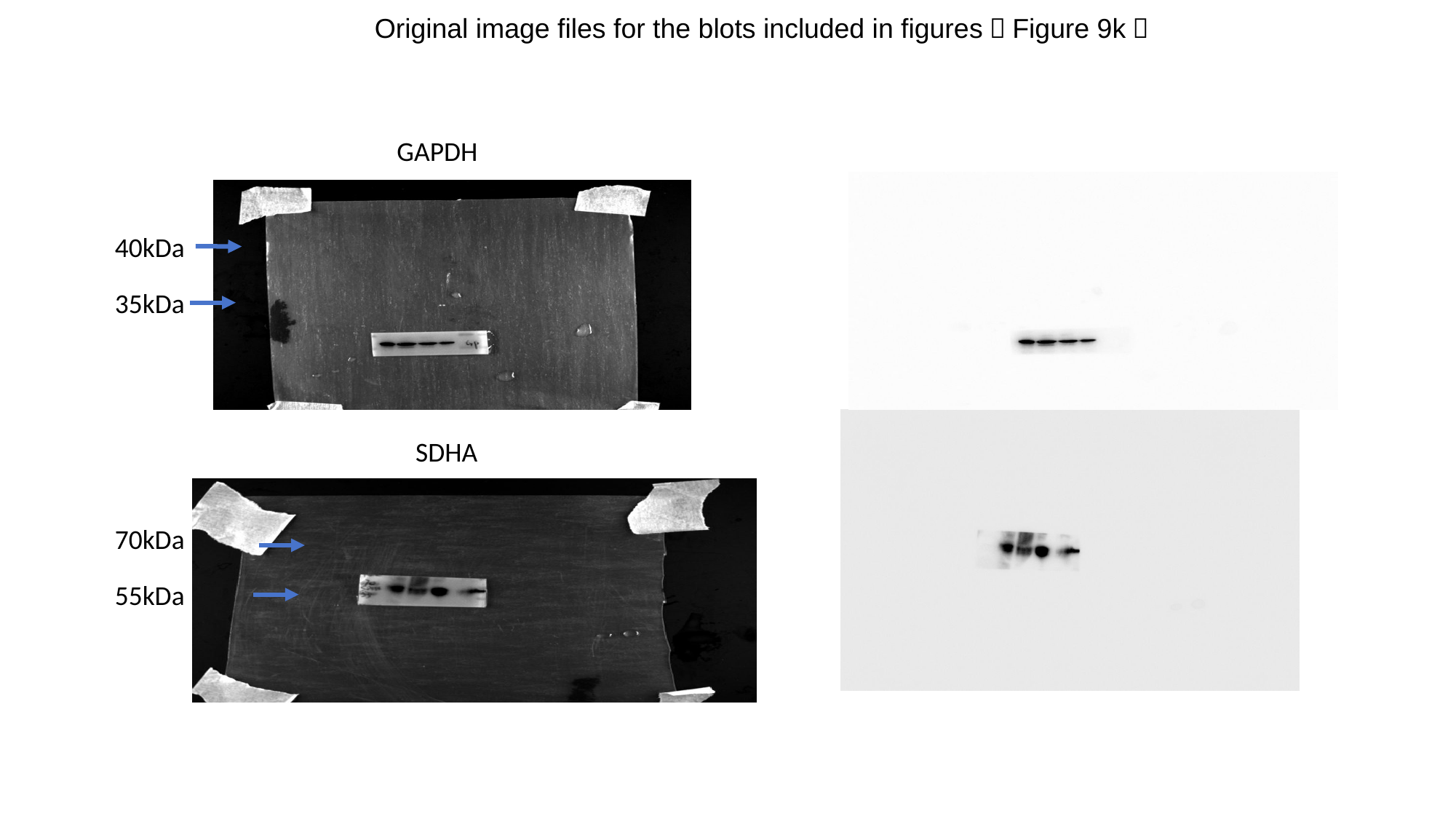

Original image files for the blots included in figures（Figure 9k）
GAPDH
40kDa
35kDa
SDHA
70kDa
55kDa
