# Supplementary material for: Identification of molecular clusters and a risk prognosis model for diffuse large B-cell lymphoma based on lactate metabolism-related genes
Source: Ann Hematol. 2025 Apr 5;104(5):2847–67. doi: 10.1007/s00277-025-06321-1 (PMC12141129; doi:10.1007/s00277-025-06321-1)
Supplement: Supplementary file 1 — Supplementary Material 1 [file 277_2025_6321_MOESM1_ESM.zip › Supplementary File20250225/Supplementary material1.docx]

**Supplementary Figures:**

**Figure S1.** Condition of prognostic LMRGs and the immune checkpoints and functional analyses within two clusters. **a.** Heatmap visualizing the different expression patterns of the 98 LMRGs in cluster 1 and cluster 2 (red: risk LMRGs, black: protective LMRGs). **b.** Obtained 98 genes based on univariate Cox regression analysis. **c.** The expressions of immune checkpoints among these two clusters. **d.** ESTIMATE algorithm. **e.** GO-BP analysis. ** *p* < 0.01, and *** *p* < 0.001, ns no significant.

| 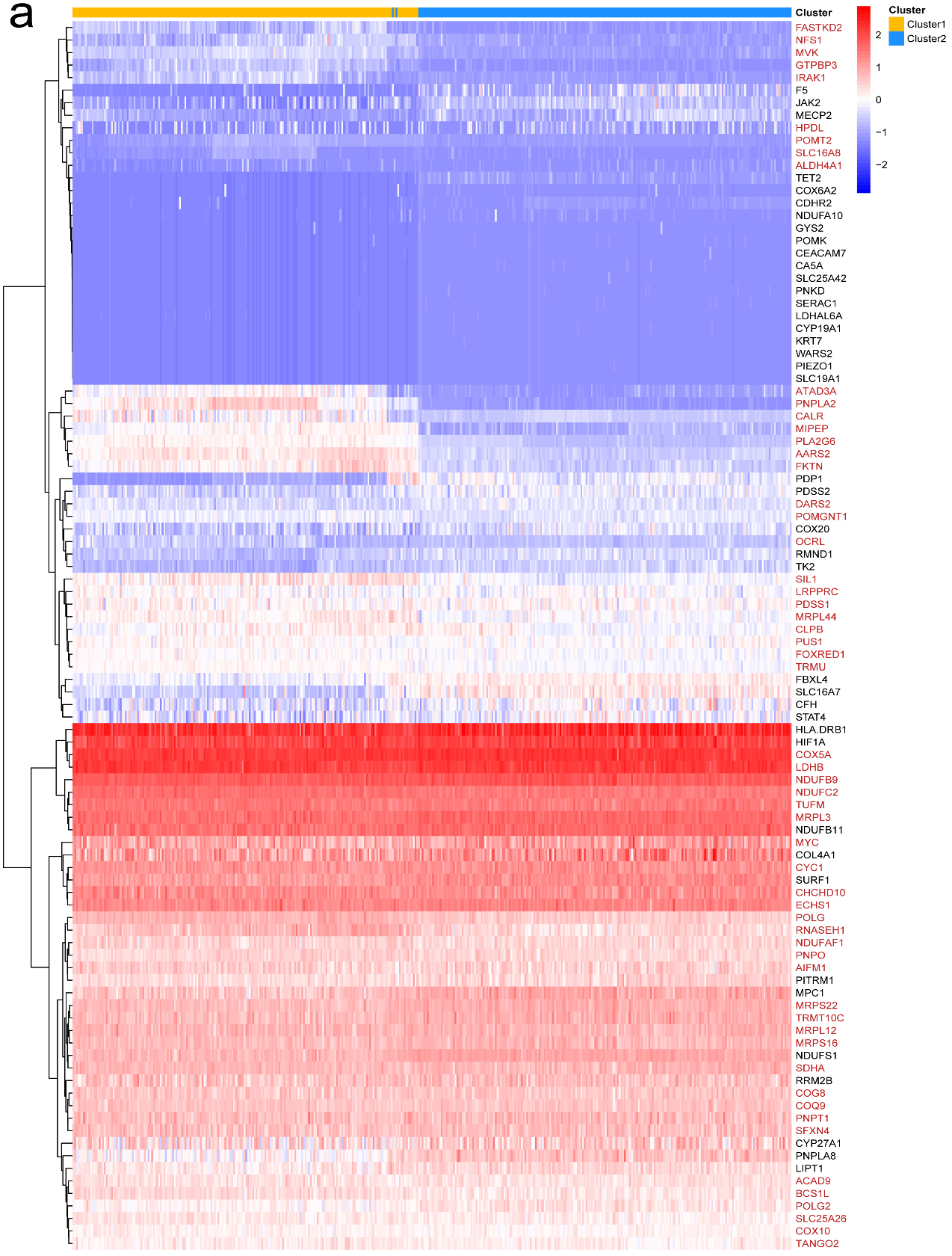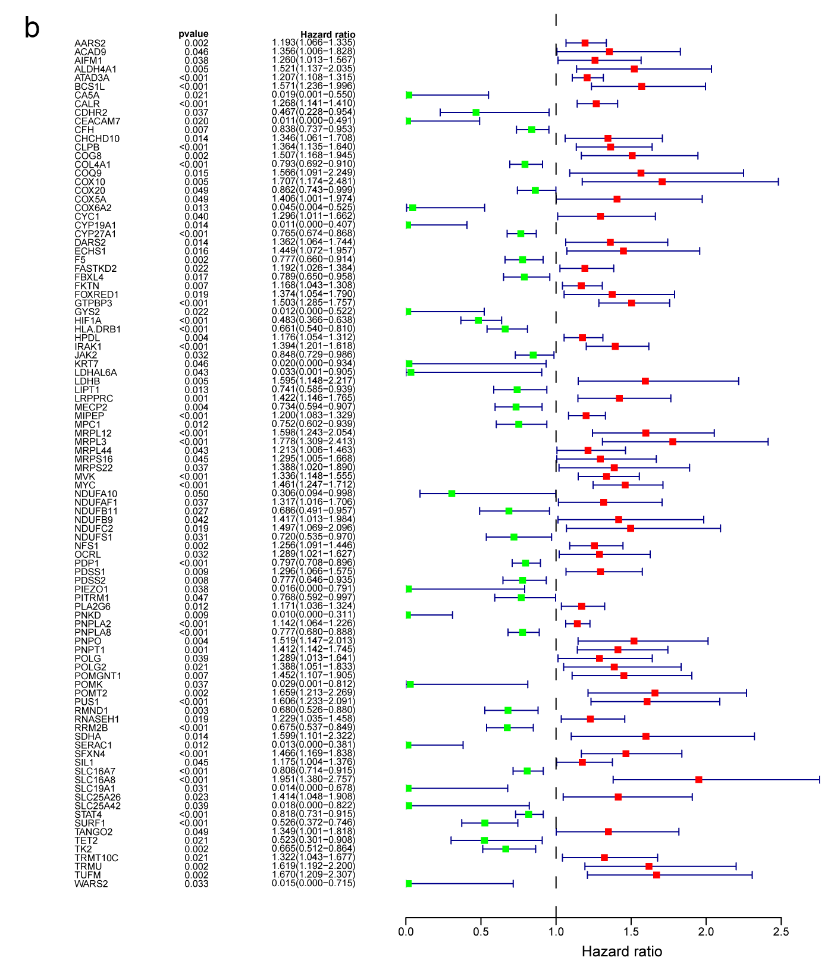  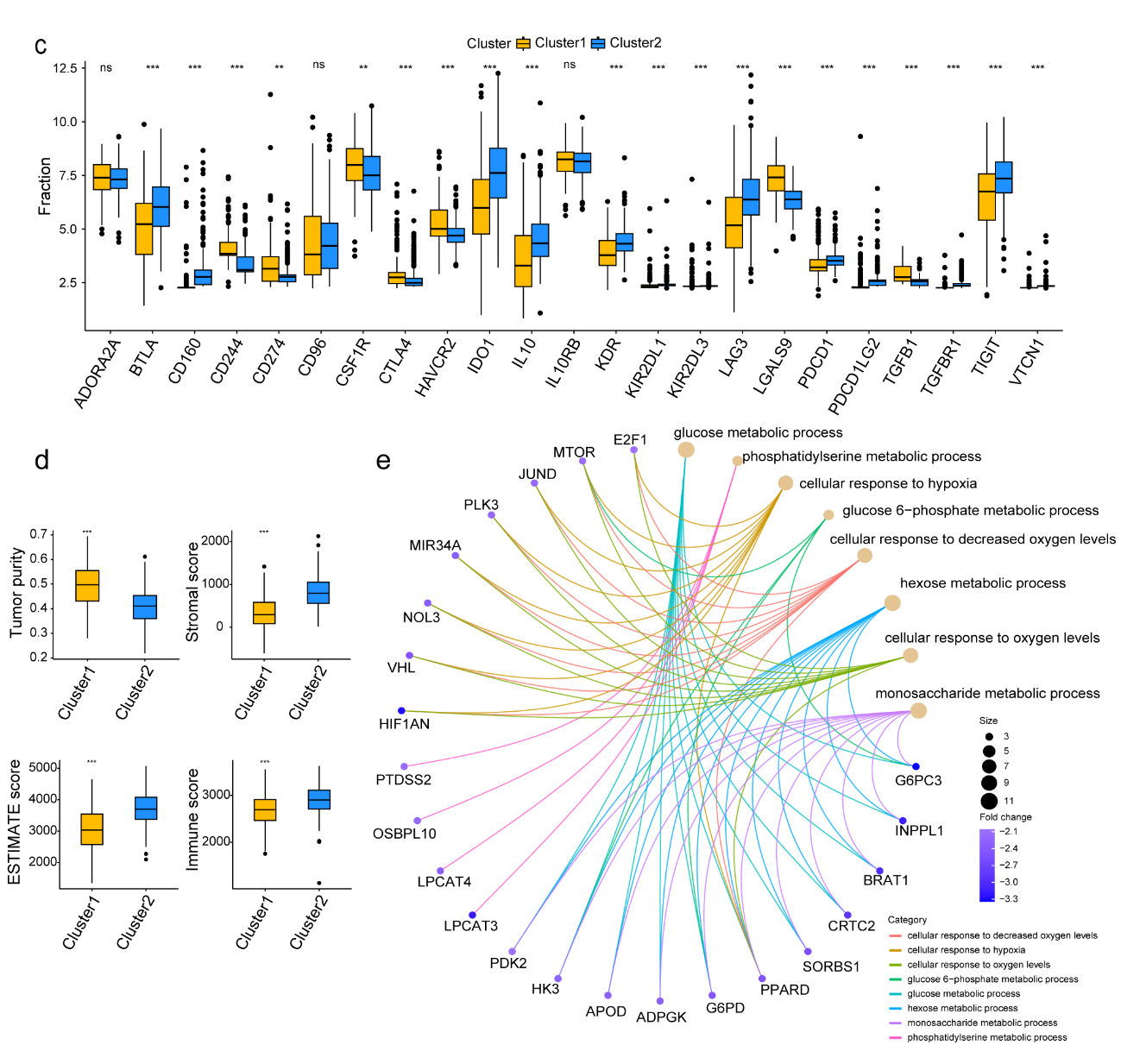 |
| --- |
|  |

**Figure S2.** a. Expression levels of *HIF1A*, *NDUFB11*, *MYC*, *PITRM1*, and *SDHA* in the high-risk and low-risk groups. b. Median expression values of *HIF1A*, *NDUFB11*, *MYC*, *PITRM1*, and *SDHA* in the training dataset. Survival analysis based on COO classification (c) and the risk scores of the different COO classifications (d) in the training group**.** *** *p* < 0.001.

| 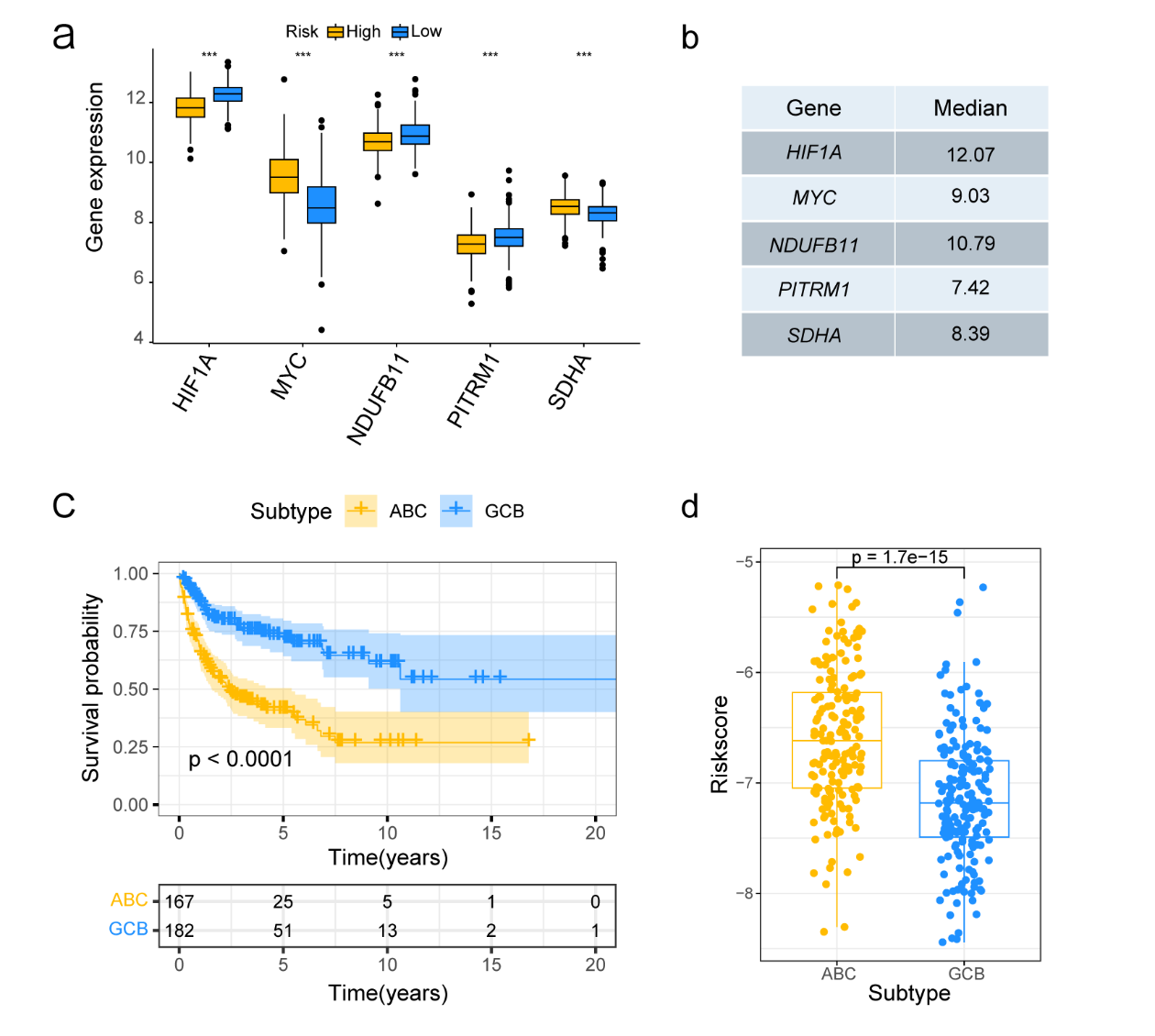 |
| --- |
|  |

**Figure S3.** **a.** Differential expression profiles of *HIF1A*, *NDUFB11*, *MYC*, *PITRM1*, and *SDHA* across high- and low-risk cohorts. **b.** Median expression values of *HIF1A*, *NDUFB11*, *MYC*, *PITRM1*, and *SDHA* in the validation dataset. **c-d.** Analyzing survival based on COO classification (c) and the risk scores associated with various COO classifications (d) within the validation group. **e-f.** Overall survival analyses between high- and low-risk groups in DLBCL patients possessed ABC-type (e) and GCB-type (f). *** *p* < 0.001, ns no significant.

| 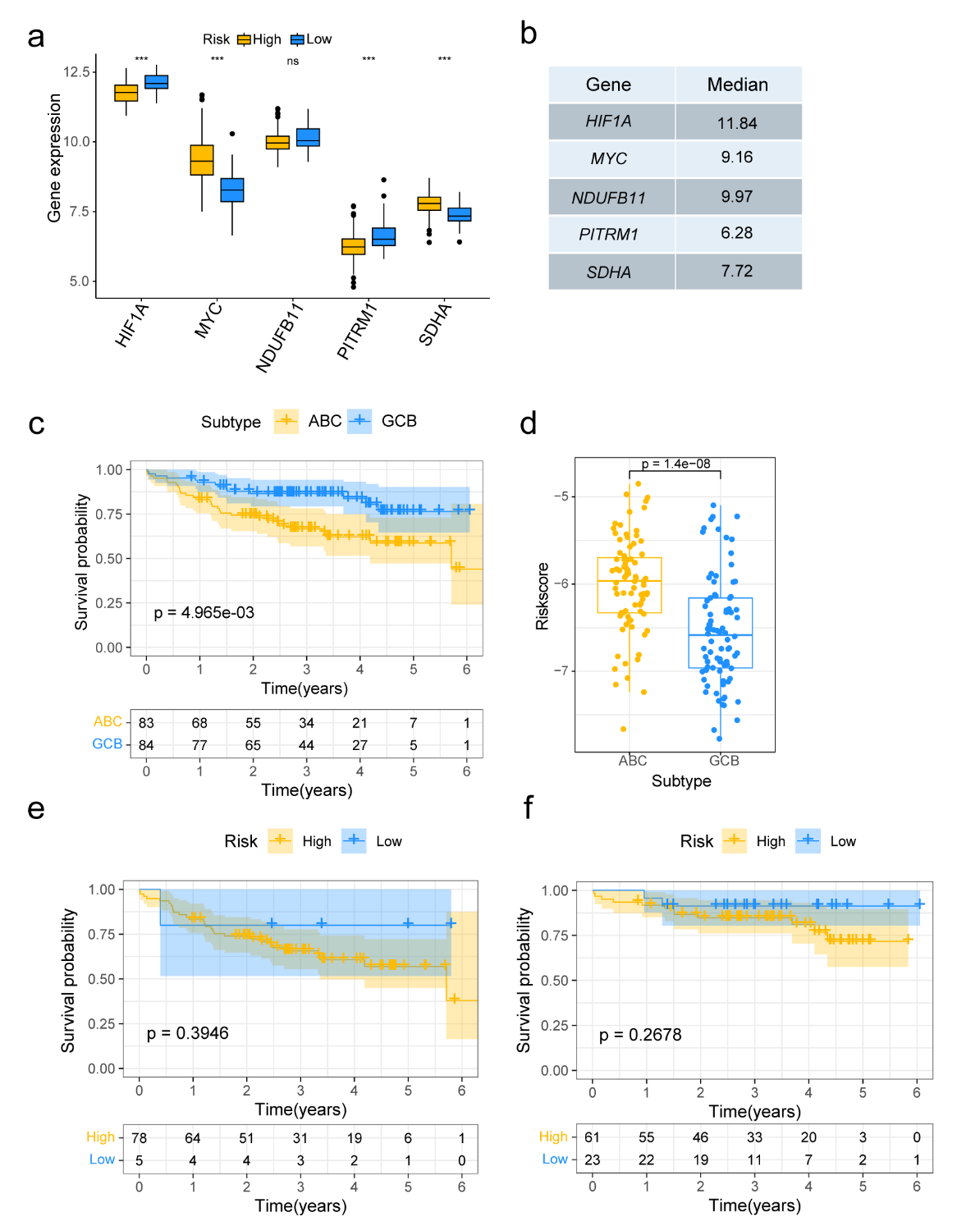 |
| --- |

| **Figure S4.** **a-b.** Heatmap expression of the clinical parameters of the two risk groups in GSE10846 (a) and GSE87371 (b). **c-g.** Associations between risk score and clinical characteristics (gender, LDH level, number of extranodal sites, stage and IPI) in the GSE10846 dataset. **h.** Associations between risk score and clinical characteristic (gender) in the GSE87371 dataset. |
| --- |

| 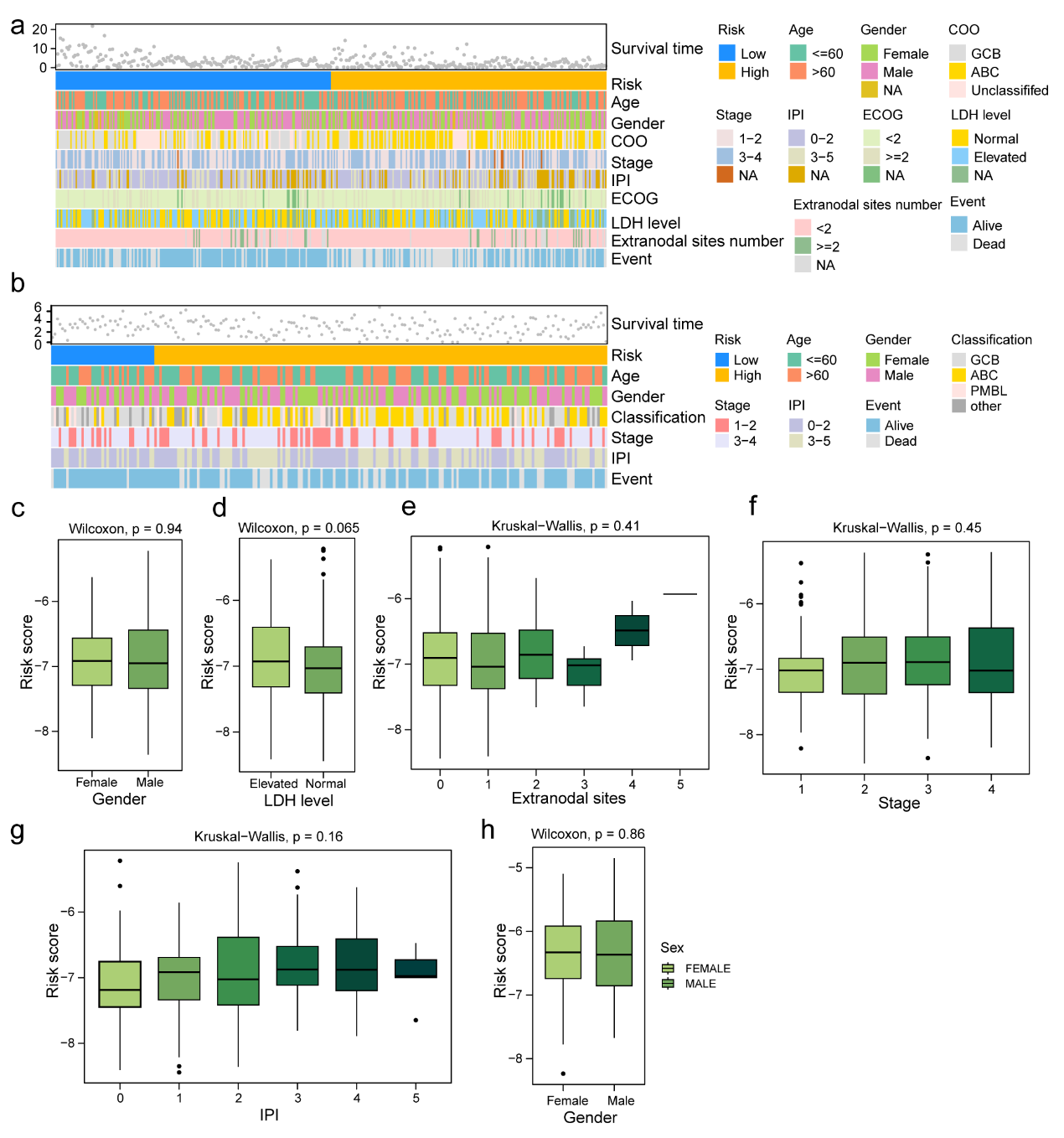 |
| --- |
|  |

| **Figure S5. a.** The correlations between risk score and diverse immune cells. **b.** The relationships between risk score and several immune checkpoint targets in DLBCL. |
| --- |
| 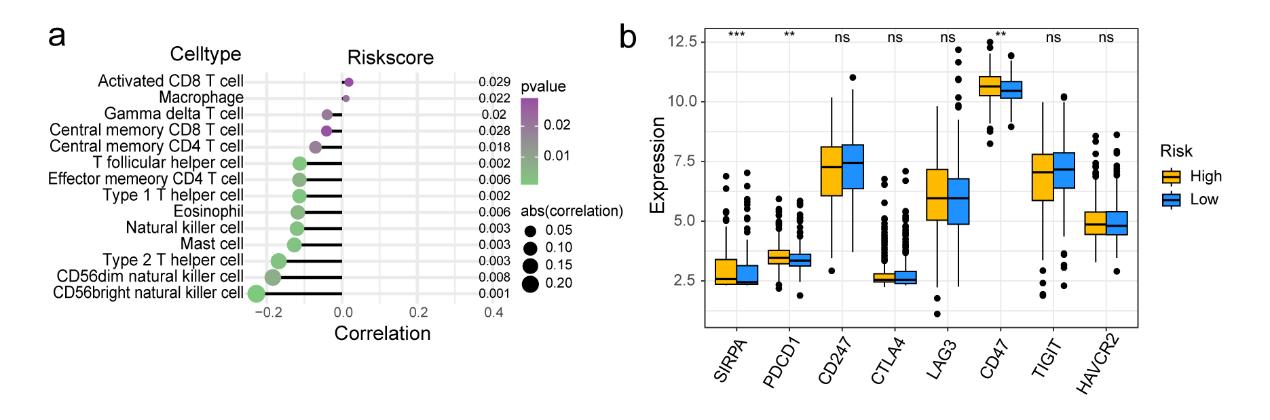 |

**Figure S6.** **a-k.** Drug sensitivity analysis of the high-risk and low-risk groups.

| 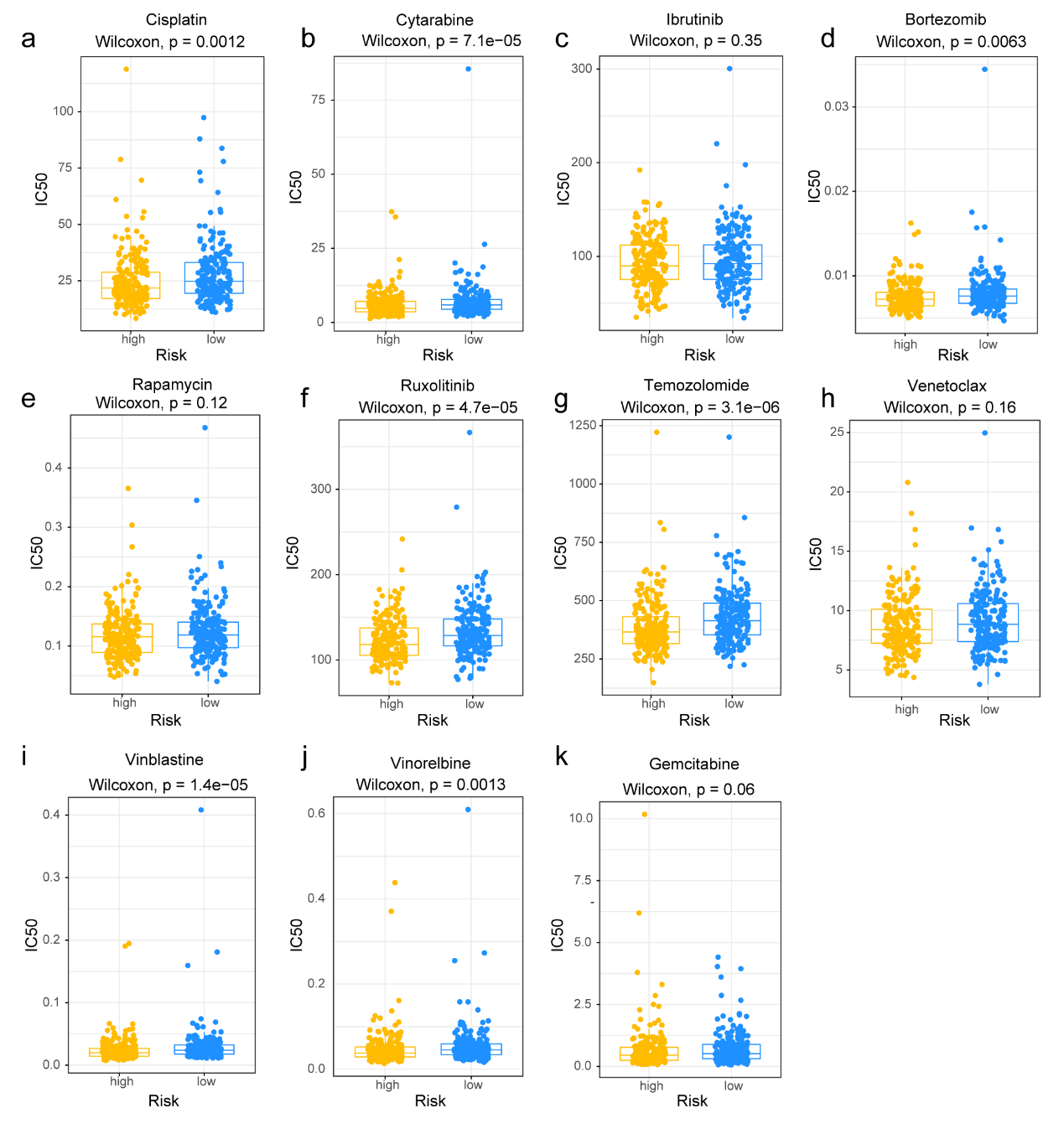 |
| --- |
|  |
