# Supplementary material for: Identification of molecular clusters and a risk prognosis model for diffuse large B-cell lymphoma based on lactate metabolism-related genes
Source: Ann Hematol. 2025 Apr 5;104(5):2847–67. doi: 10.1007/s00277-025-06321-1 (PMC12141129; doi:10.1007/s00277-025-06321-1)
Supplement: Supplementary file 1 — Supplementary Material 1 [file 277_2025_6321_MOESM1_ESM.zip › Supplementary File20250225/Supplementary material2.docx]

**Table S1.** Clinicopathological features of DLBCL patients in the GSE10846 and GSE87371.

| Characteristic | GSE10846 | Characteristic | GSE87371 |
| --- | --- | --- | --- |
| Age, n (%) | | Age, n (%) | |
| ≤60 | 188 (45.6%) | ≤60 | 115 (52.0%) |
| >60 | 224 (54.4%) | >60 | 106 (48.0%) |
| Gender, n (%) |  | Gender, n (%) | |
| Female | 172 (41.7%) | Female | 105 (47.5%) |
| Male | 222 (53.9%) | Male | 116 (52.5%) |
| NA | 18 (4.4%) | Category, n (%) |  |
| ECOG, n (%) | | GCB | 84 (38.0%) |
| <2 | 295 (71.6%) | ABC | 83 (37.6%) |
| ≥2 | 93 (22.6%) | PMBL | 20 (9.0%) |
| NA | 24 (5.8%) | other | 34 (15.4%) |
| Ann Arbor stage, n (%) | | Ann Arbor stage, n (%) | |
| Ⅰ-Ⅱ | 188 (45.6%) | Ⅰ-Ⅱ | 71 (32.1%) |
| Ⅲ-Ⅳ | 217 (52.7%) | Ⅲ-Ⅳ | 150 (67.9%) |
| NA | 7 (1.7%) | IPI, n (%) |  |
| LDH level, n (%) | | 0-2 | 119 (53.8%) |
| Normal | 173 (42.0%) | 3-5 | 102 (46.2%) |
| Elevated | 177 (43.0%) | |  |
| NA | 62 (15.0%) |  |  |
| Extranodal sites, n (%) | | |  |
| <2 | 351 (85.2%) | |  |
| ≥2 | 30 (7.3%) |  |  |
| NA | 31 (7.5%) |  |  |
| COO, n (%) | |  | |
| GCB | 182 (44.2%) |  |  |
| ABC | 167 (40.5%) |  |  |
| Unclassified | 63 (15.3%) |  |  |

Abbreviations: NA: not available, ECOG: Eastern Cooperative Oncology Group, COO: cell-of-origin, GCB: germinal center B-cell-like, ABC: activated B-cell-like, LDH: lactate dehydrogenase, PMBL: primary mediastinal large B-cell lymphoma, IPI: international prognostic index

**Table S2 .** Patient characteristics and cluster stratification in GSE10846.

| **Characteristic** | **GSE10846** | | ***p*-value** |
| --- | --- | --- | --- |
|  | **Cluster1**  **n=196** | **Cluster2**  **n=216** |  |
| Age, n (%) |  |  | 0.095 |
| ≤60 | 81 (41%) | 107 (50%) |  |
| >60 | 115 (59%) | 109 (50%) |  |
| Gender, n (%) |  |  | <0.001 |
| Female | 80 (41%) | 92 (43%) |  |
| Male | 98 (50%) | 124 (57%) |  |
| NA | 18 (9.2%) | 0 (0%) |  |
| ECOG, n (%) |  |  | <0.001 |
| <2 | 152 (78%) | 143 (66%) |  |
| ≥2 | 41 (21%) | 52 (24%) |  |
| NA | 3 (1.5%) | 21 (9.7%) |  |
| Ann Arbor stage, n (%) |  |  | 0.200 |
| Ⅰ-Ⅱ | 90 (46%) | 98 (45%) |  |
| Ⅲ-Ⅳ | 105 (54%) | 112 (52%) |  |
| NA | 1 (0.5%) | 6 (2.8%) |  |
| LDH level, n (%) |  |  | 0.040 |
| Normal | 82 (42%) | 91 (42%) |  |
| Elevated | 93 (47%) | 84 (39%) |  |
| NA | 21 (11%) | 41 (19%) |  |
| Extranodal sites, n (%) |  |  | <0.001 |
| <2 | 195 (99%) | 156 (72%) |  |
| ≥2 | 0 (0%) | 30 (14%) |  |
| NA | 1 (0.5%) | 30 (14%) |  |
| COO, n (%) |  |  | >0.900 |
| GCB | 85 (43%) | 97 (45%) |  |
| ABC | 81 (41%) | 86 (40%) |  |
| Unclassified | 30 (15%) | 33 (15%) |  |
| Futime, median (Q1, Q3) | 2.93 (1.01, 6.55) | 1.95 (0.85, 3.37) | <0.001 |

Abbreviations: NA: not available, ECOG: Eastern Cooperative Oncology Group, COO: cell-of-origin, GCB: germinal center B-cell-like, ABC: activated B-cell-like, LDH: lactate dehydrogenase, IPI: international prognostic index

**Table S3 .** Patient characteristics and lactate metabolism-associated model risk stratification in the training and validation groups.

| **Characteristic** | **GSE10846** | | ***p*-value** | **Characteristic** | **GSE87371** | | ***p*-value** |
| --- | --- | --- | --- | --- | --- | --- | --- |
|  | **High risk**  **n=206** | **Low risk**  **n=206** |  |  | **High risk**  **n=180** | **Low risk**  **n=41** |  |
| Age, n (%) |  |  | 0.075 | Age |  |  | 0.400 |
| ≤60 | 85 (41%) | 103 (50%) |  | <=60 | 91 (51%) | 24 (59%) |  |
| >60 | 121 (59%) | 103 (50%) |  | >60 | 89 (49%) | 17 (41%) |  |
| Gender, n (%) |  |  | 0.600 | Gender |  |  | 0.400 |
| Female | 88 (43%) | 84 (41%) |  | Female | 88 (49%) | 17 (41%) |  |
| Male | 111 (54%) | 111 (54%) |  | Male | 92 (51%) | 24 (59%) |  |
| NA | 7 (3.4%) | 11 (5.3%) |  | Category |  |  | <0.001 |
| ECOG, n (%) |  |  | 0.200 | GCB | 61 (34%) | 23 (56%) |  |
| <2 | 139 (67%) | 156 (76%) |  | ABC | 78 (43%) | 5 (12%) |  |
| ≥2 | 53 (26%) | 40 (19%) |  | PMBL | 16 (8.9%) | 4 (9.8%) |  |
| NA | 14 (6.8%) | 10 (4.9%) |  | Other | 25 (14%) | 9 (22%) |  |
| Ann Arbor stage, n (%) |  |  | 0.200 | Ann Arbor stage, n (%) | |  | >0.900 |
| Ⅰ-Ⅱ | 93 (45%) | 95 (46%) |  | 1_2 | 58 (32%) | 13 (32%) |  |
| Ⅲ-Ⅳ | 107 (52%) | 110 (53%) |  | 3_4 | 122 (68%) | 28 (68%) |  |
| NA | 6 (2.9%) | 1 (0.5%) |  | IPI |  |  | 0.016 |
| LDH level, n (%) |  |  | 0.200 | 0_2 | 90 (50%) | 29 (71%) |  |
| Normal | 79 (38%) | 94 (46%) |  | 3_5 | 90 (50%) | 12 (29%) |  |
| Elevated | 90 (44%) | 87 (42%) |  |  |  |  |  |
| NA | 37 (18%) | 25 (12%) |  |  |  |  |  |
| Extranodal sites, n (%) |  |  | >0.900 |  |  |  |  |
| <2 | 176 (85%) | 175 (85%) |  |  |  |  |  |
| ≥2 | 15 (7.3%) | 15 (7.3%) |  |  |  |  |  |
| NA | 15 (7.3%) | 16 (7.8%) |  |  |  |  |  |
| COO, n (%) |  |  | <0.001 |  |  |  |  |
| GCB | 62 (30%) | 120 (58%) |  |  |  |  |  |
| ABC | 118 (57%) | 49 (24%) |  |  |  |  |  |
| Unclassified | 26 (13%) | 37 (18%) |  |  |  |  |  |

Abbreviations: NA: not available, ECOG: Eastern Cooperative Oncology Group, COO: cell-of-origin, GCB: germinal center B-cell-like, ABC: activated B-cell-like, LDH: lactate dehydrogenase, PMBL: primary mediastinal large B-cell lymphoma, IPI: international prognostic index
