# Supplementary material for: Identification of molecular clusters and a risk prognosis model for diffuse large B-cell lymphoma based on lactate metabolism-related genes
Source: Ann Hematol. 2025 Apr 5;104(5):2847–67. doi: 10.1007/s00277-025-06321-1 (PMC12141129; doi:10.1007/s00277-025-06321-1)
Supplement: Supplementary file 1 — Supplementary Material 1 [file 277_2025_6321_MOESM1_ESM.zip › Supplementary File20250225/Supplementary material4.docx]

**Table S5.** Information of antibodies involved.

| Antigens | source | Identifier |
| --- | --- | --- |
| Anti-GAPDH | Proteintech | Cat#60004-1-Ig |
| Anti-SDHA | Proteintech | Cat#14865-1-AP |
