# Supplementary material for: Identification of molecular clusters and a risk prognosis model for diffuse large B-cell lymphoma based on lactate metabolism-related genes
Source: Ann Hematol. 2025 Apr 5;104(5):2847–67. doi: 10.1007/s00277-025-06321-1 (PMC12141129; doi:10.1007/s00277-025-06321-1)
Supplement: Supplementary file 1 — Supplementary Material 1 [file 277_2025_6321_MOESM1_ESM.zip › Supplementary File20250225/Supplementary material5.docx]

**Table S6.** List of sequences for siRNA.

| siRNA |  | Sequence (5′-3′) |
| --- | --- | --- |
| si-SDHA | Sense | GGUGCUGGUUGUCUCAUUA |
|  | Antisense | UAAUGAGACAACCAGCACC |
| si-control | Sense | UUCUCCGAACGUGUCACGU |
|  | Antisense | ACGUGACACGUUCGGAGAA |
