# Supplementary figures and images for: Identification of molecular clusters and a risk prognosis model for diffuse large B-cell lymphoma based on lactate metabolism-related genes
Source: Ann Hematol. 2025 Apr 5;104(5):2847–67. doi: 10.1007/s00277-025-06321-1 (PMC12141129; doi:10.1007/s00277-025-06321-1)

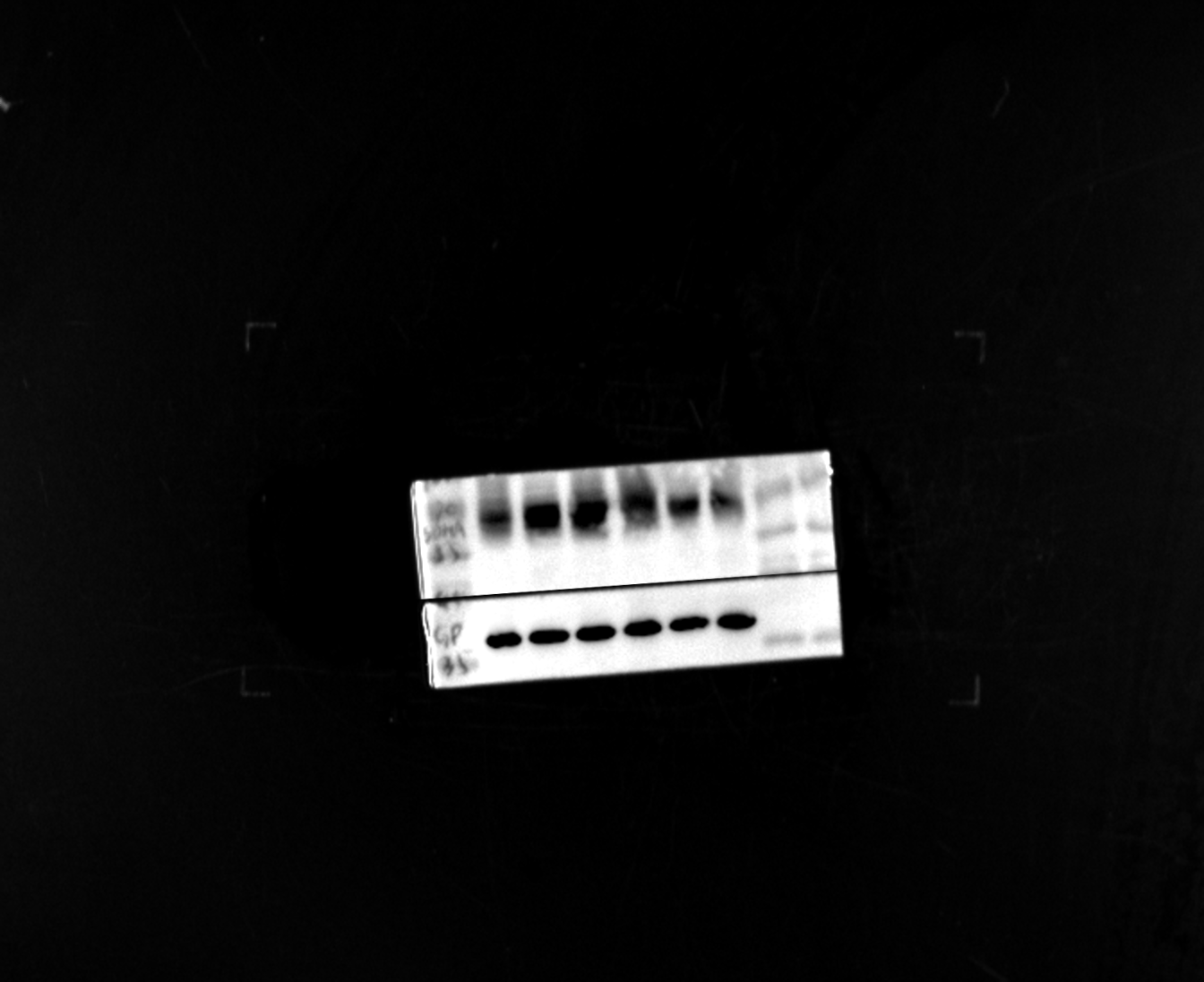

Supplement: Supplementary file 1 — Supplementary Material 1 [file 277_2025_6321_MOESM1_ESM.zip › Supplementary File20250225/Original Images for Blots_Gels/(Membrane)_raw.tif]

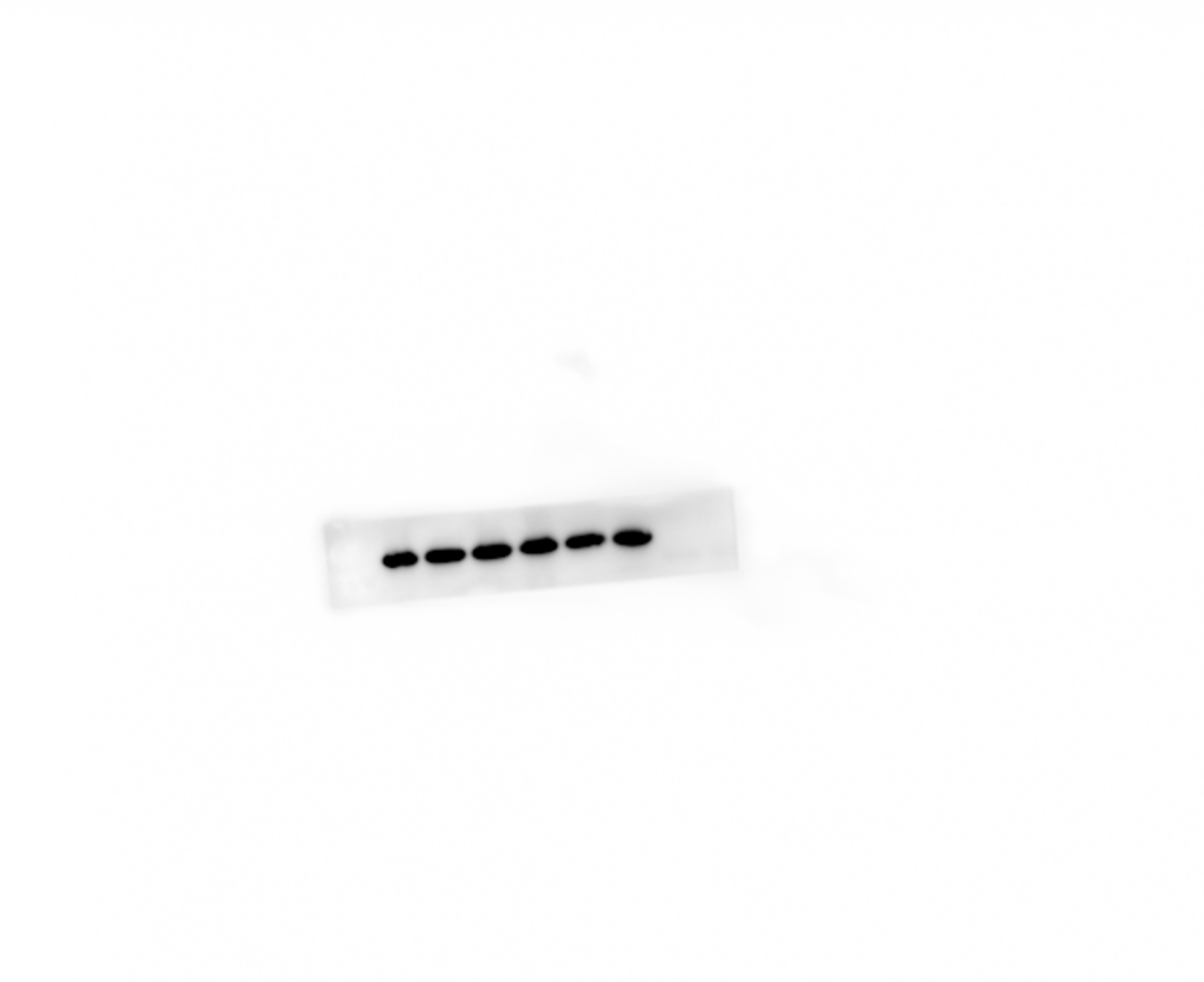

Supplement: Supplementary file 1 — Supplementary Material 1 [file 277_2025_6321_MOESM1_ESM.zip › Supplementary File20250225/Original Images for Blots_Gels/GAPDH(Chemi).Tif]

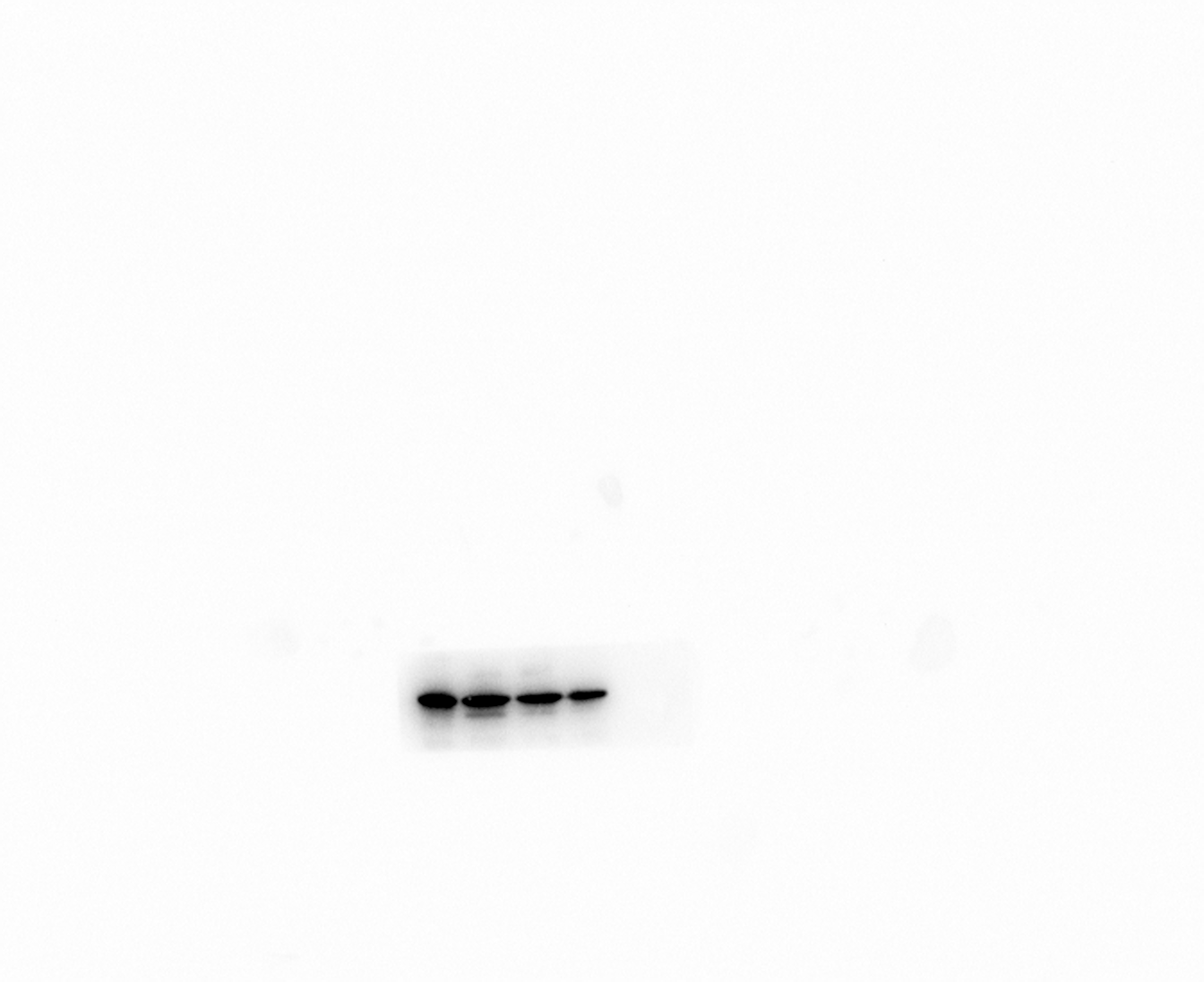

Supplement: Supplementary file 1 — Supplementary Material 1 [file 277_2025_6321_MOESM1_ESM.zip › Supplementary File20250225/Original Images for Blots_Gels/GAPDH(Chemi)_siSDHA.Tif]

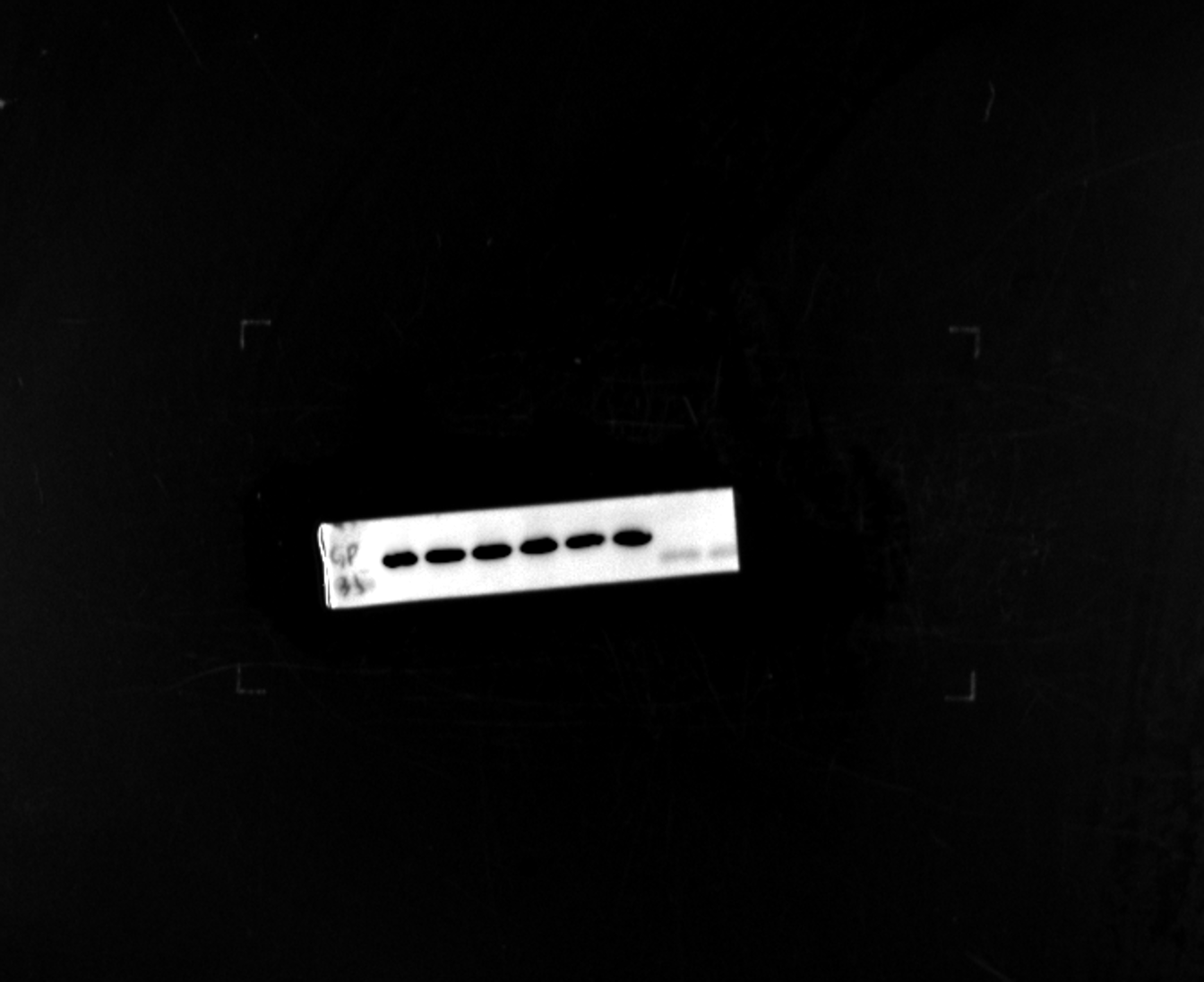

Supplement: Supplementary file 1 — Supplementary Material 1 [file 277_2025_6321_MOESM1_ESM.zip › Supplementary File20250225/Original Images for Blots_Gels/GAPDH(Overlay).Tif]

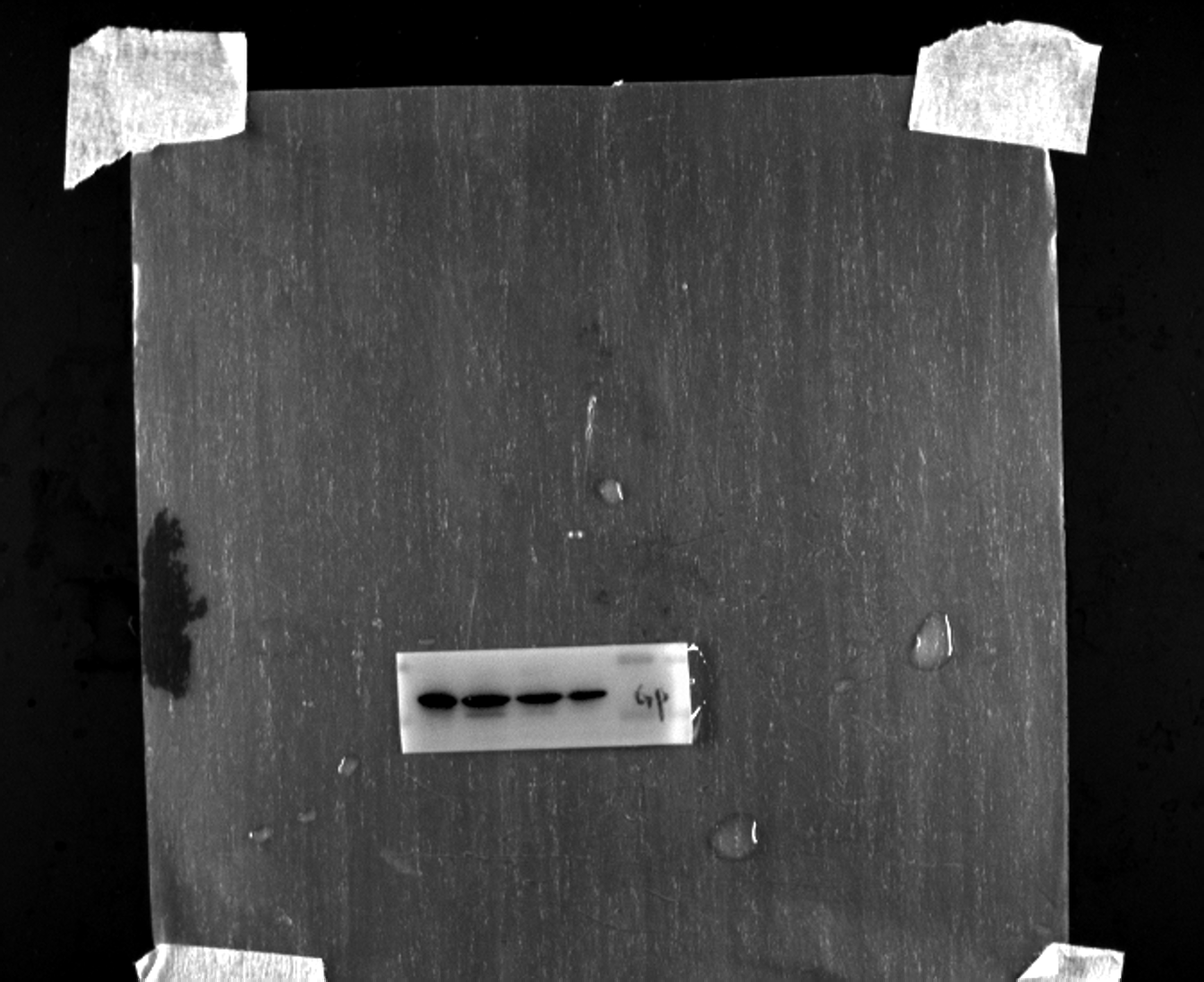

Supplement: Supplementary file 1 — Supplementary Material 1 [file 277_2025_6321_MOESM1_ESM.zip › Supplementary File20250225/Original Images for Blots_Gels/GAPDH(Overlay)_siSDHA.Tif]

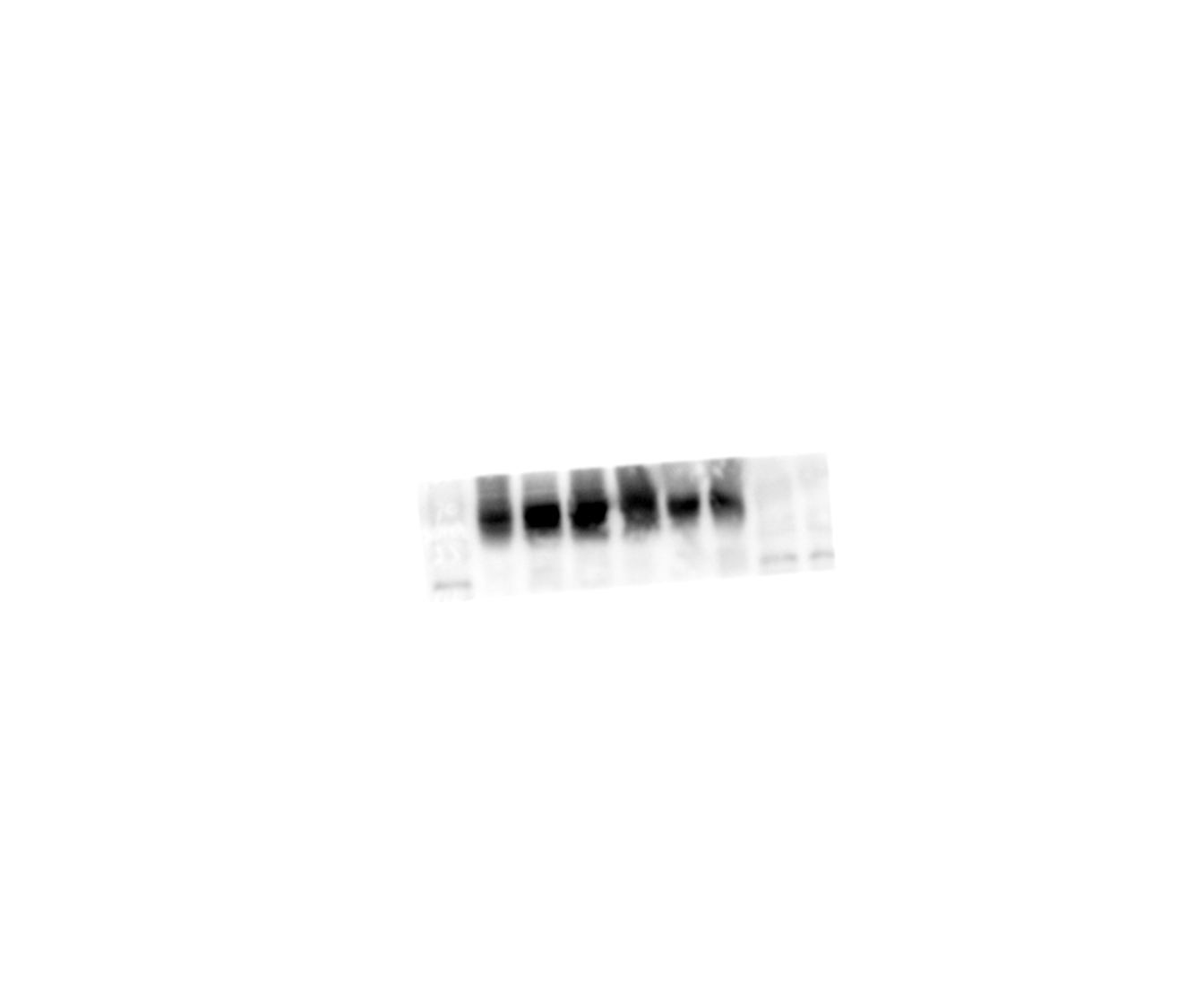

Supplement: Supplementary file 1 — Supplementary Material 1 [file 277_2025_6321_MOESM1_ESM.zip › Supplementary File20250225/Original Images for Blots_Gels/SDHA(Chemi).Tif]

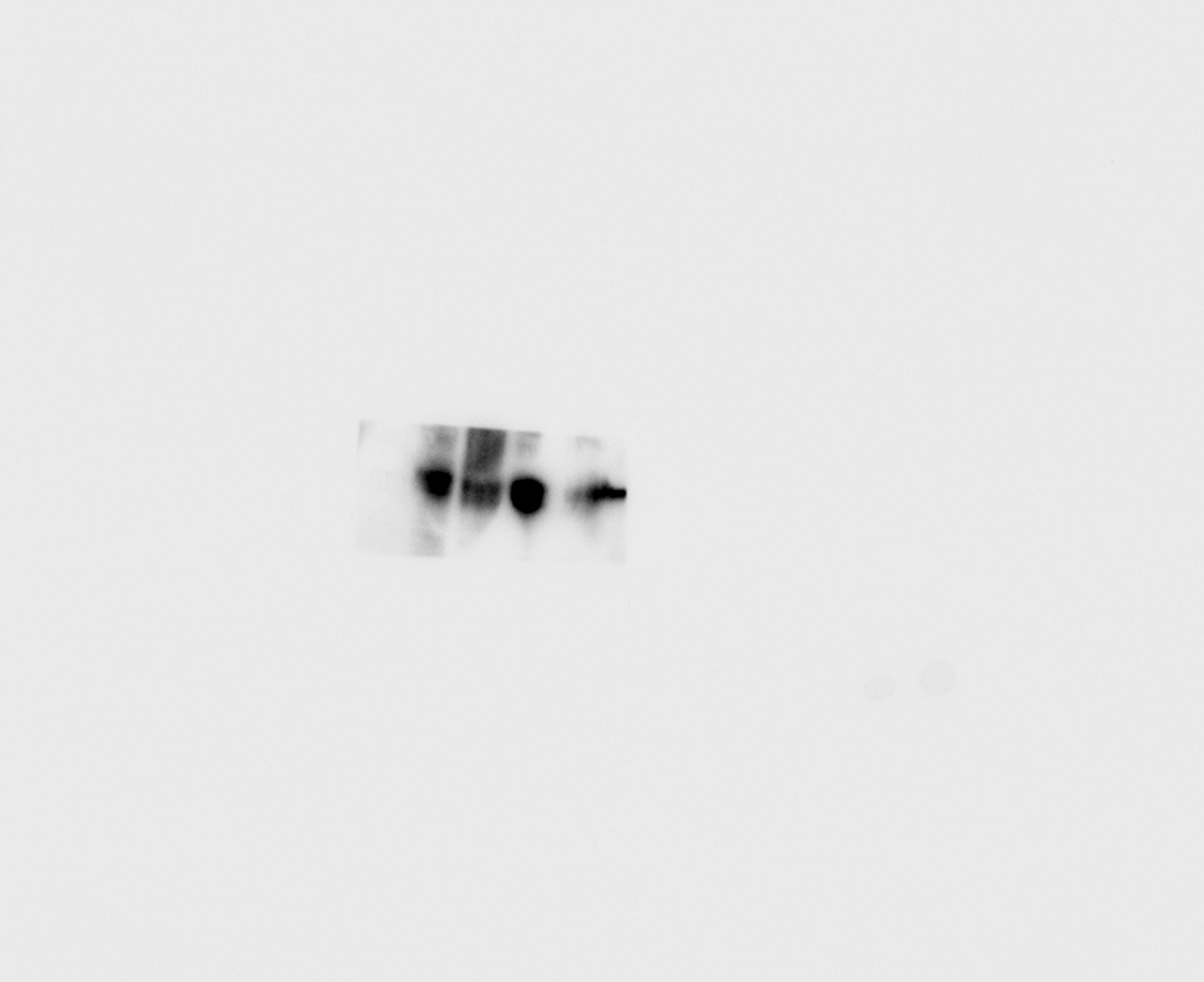

Supplement: Supplementary file 1 — Supplementary Material 1 [file 277_2025_6321_MOESM1_ESM.zip › Supplementary File20250225/Original Images for Blots_Gels/SDHA(Chemi)_siSDHA.Tif]

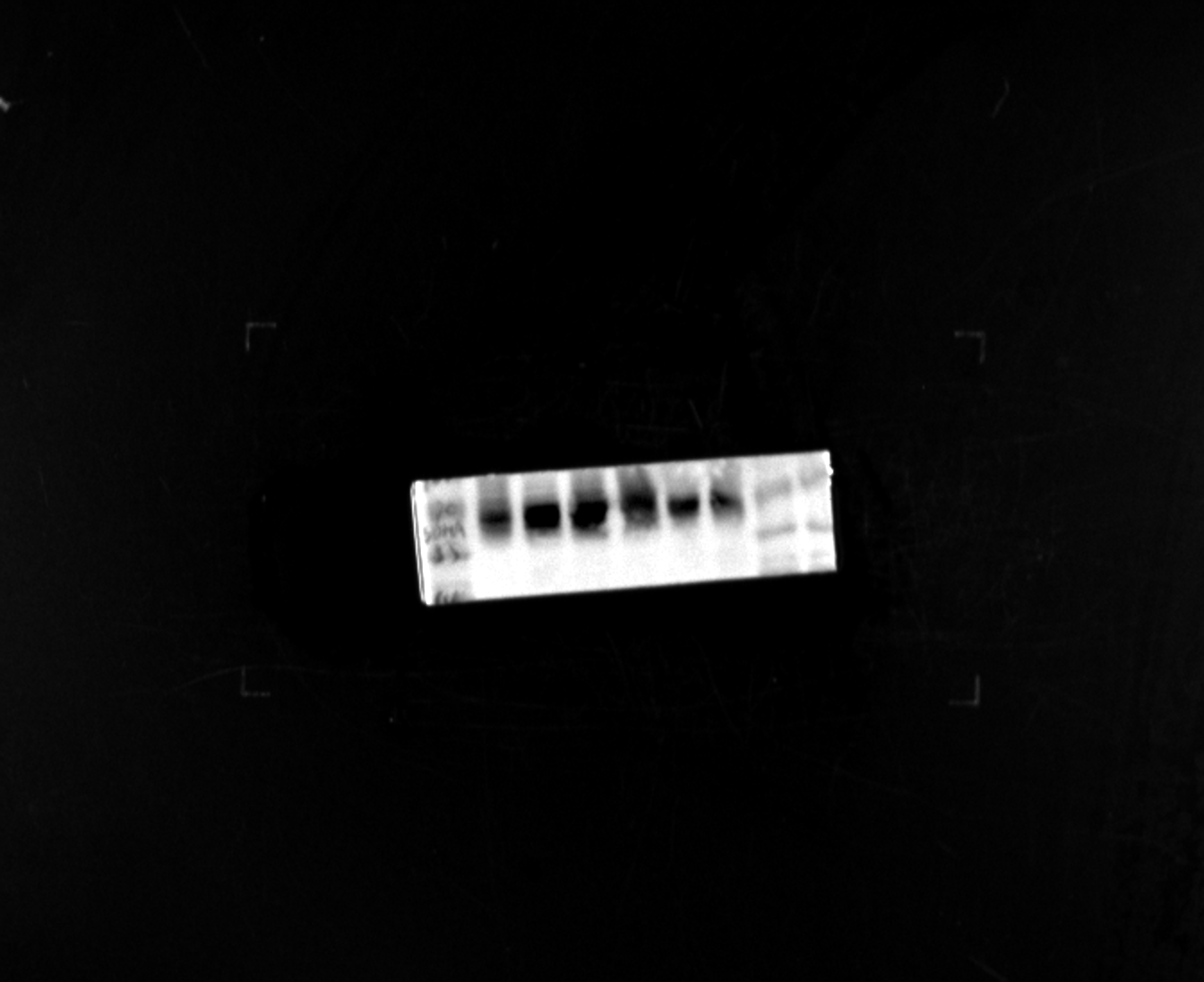

Supplement: Supplementary file 1 — Supplementary Material 1 [file 277_2025_6321_MOESM1_ESM.zip › Supplementary File20250225/Original Images for Blots_Gels/SDHA(Overlay).Tif]

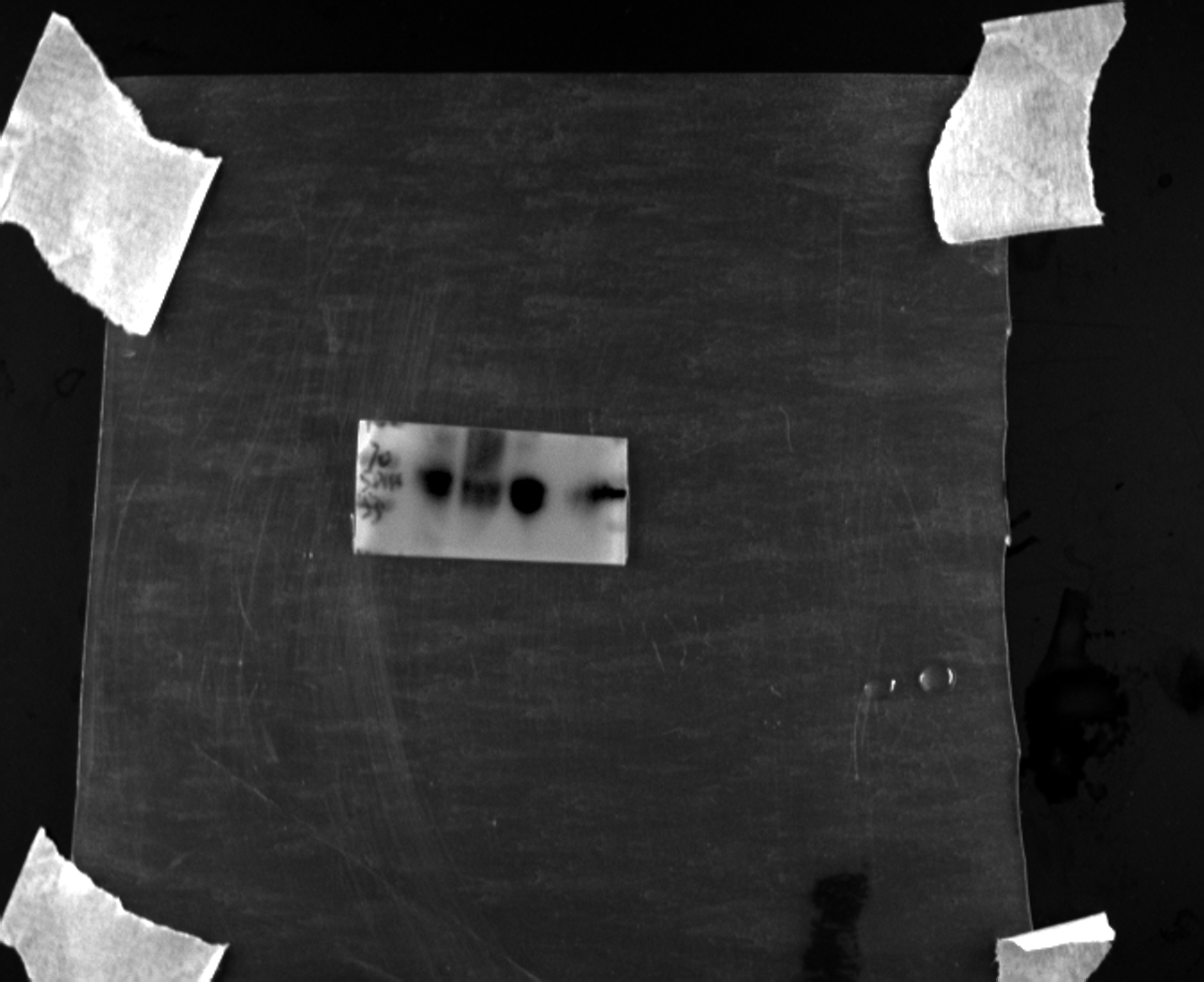

Supplement: Supplementary file 1 — Supplementary Material 1 [file 277_2025_6321_MOESM1_ESM.zip › Supplementary File20250225/Original Images for Blots_Gels/SDHA(Overlay)_siSDHA.Tif]
